# Association of body mass index trajectory and hypertension risk: A systematic review of cohort studies and network meta-analysis of 89,094 participants

**DOI:** 10.3389/fcvm.2022.941341

**Published:** 2023-01-04

**Authors:** Ling Tan, Lin-zi Long, Xiao-chang Ma, Wen-wen Yang, Fei-fei Liao, Yu-xuan Peng, Jie-ming Lu, A-ling Shen, Dong-qing An, Hua Qu, Chang-geng Fu

**Affiliations:** ^1^Xiyuan Hospital, China Academy of Chinese Medical Sciences, Beijing, China; ^2^National Cardiovascular Clinical Medical Research Center of TCM, Beijing, China; ^3^Graduate School of Beijing University of Chinese Medicine, Beijing, China; ^4^Academy of Integrative Medicine, Fujian University of Traditional Chinese Medicine, Fuzhou, Fujian, China; ^5^Affiliated Hospital of Traditional Chinese Medicine, Xinjiang Medical University, Ürümqi, China

**Keywords:** body mass index trajectory, hypertension, growth trajectory, network meta-analysis, high blood pressure

## Abstract

**Introduction:**

Body mass index (BMI) trajectories, such as non-linear time trends and nonlinear changes in BMI with age, can provide information on the underlying temporal health patterns. The relationship between BMI trajectories and the risk of hypertension remains controversial.

**Methods:**

PubMed, Embase, Cochrane, Scopus, and Web of Science databases were searched from their inception to January 31, 2022. We categorized BMI trajectories as “Stable high,” “table normal,” “Stable low,” “Fluctuated (sharp increase),” and “Fluctuated (elevated-decrease).” The main outcome was the relative risk for the prevalence of hypertension in the different BMI trajectories. Potential sources of heterogeneity were examined using meta-regression and subgroup analysis. A publication bias test and Grading of Recommendations Assessment, Development and Evaluation (GRADE) approach were also used.

**Results:**

The 18 cohort studies included 89,094 participants. Compared with the “Stable normal” trajectory, “Stable high,” “Fluctuated (sharp increase),” and “Fluctuated (elevated-decrease)” trajectories were associated with an increased relative risk of hypertension: [RR (95% CI)]: 1.80 (1.29 2.50), *p* < 0.001; 1.53 (1.27 1.83), *p* < 0.001; 1.30 (1.24 1.37), *p* = 0.001, respectively. The “Stable low” trajectory was associated with a reduced risk of hypertension [0.83 (0.79 0.83), *p* < 0.001]. The “Stable high” trajectory (surface under the cumulative ranking curve = 88.1%) had the highest probability of developing hypertension in the population. The certainty of the evidence for direct comparisons of the incidence of hypertension between various BMI trajectories was generally very low.

**Conclusion:**

Our findings suggested that “Stable high,” “Fluctuated (sharp increase),” and “Fluctuated (elevated-decrease)” trajectories were associated with an increased relative risk of hypertension, with the “Stable high” trajectory most likely associated with hypertension.

**Systematic review registration:**

[https://www.crd.york.ac.uk/prospero/display_record.php?RecordID=308575], identifier [CRD42022308575].

## 1. Introduction

Hypertension is a clinical syndrome characterised by elevated systemic arterial blood pressure (systolic blood pressure ≥140 mmHg and/or diastolic blood pressure ≥90 mmHg). Hypertension may be accompanied by functional or organic damage to the heart, brain, kidney, and other organs (1). The total number of patients with hypertension in adults ≥20 years of age will likely exceed 1.6 billion by 2025 (2).

A dose-response meta-analysis of more than 2.3 million participants indicated an obvious relationship between body mass index (BMI) and the risk of hypertension (3). Although the impact of BMI on hypertension has become an increasingly important public health concern (4, 5), BMI at a single time point does not accurately predict hypertension in later life. BMI may change for a variety of reasons, and its effect on outcomes may change accordingly. Therefore, there has been growing interest in BMI trajectories because of the ability of a trajectory to capture non-linear developmental patterns of BMI over time. Ahanchi et al. (6) reported that the trend of increasing BMI with age was a better predictor of the risk of developing hypertension than a higher baseline BMI. Thus, assessing the trajectory of BMI dynamics over time can predict hypertension risk better than BMI level.

There is still some controversy regarding the relationship between various BMI trajectories and the risk of hypertension. Therefore, there is an urgent need to analyses and address these issues. Furthermore, there is no comprehensive systematic review or meta-analysis to conclude a relationship between BMI trajectory and the risk of hypertension and to clarify whether different types of BMI trajectories differ in their risk of hypertension. The present study sought to elucidate the relationship between different BMI trajectories and the risk of hypertension through a comprehensive systematic review combined with a network meta-analysis. The findings would be useful for the clinical prevention and treatment of hypertension from the perspective of BMI control.

## 2. Methods

### 2.1. Systematic literature review and selection of studies

This meta-analysis was conducted in accordance with the guidelines provided by the Preferred Reporting Items for Systematic Reviews and Meta-Analyses (PRISMA) report (7) (Supplementary Table 1). The protocol for this systematic review and meta-analysis was registered at PROSPERO (CRD202208575) before data extraction. The aim of the literature search was to identify publications on BMI trajectories and hypertension. Two reviewers (LT and HQ) independently performed a systematic search of PubMed, Cochrane Library, Embase, Scopus, and Web of Science from inception to 31 January 2022. The detailed search strategies are listed in Supplementary Table 2. Searches were not restricted by language.

Concerning the eligibility criteria, the studies: (1) had a cohort design (prospective and retrospective cohort studies), (2) reported the participants without hypertension at baseline, (3) measured BMI as the exposure and recorded the statistical method of optimal BMI trajectory classification, (4) clearly defined hypertension as the outcome, and (5) reported the number of participants and individuals with hypertension, and adjusted effect estimates as relative risk (RR), hazard ratio (HR), or odds ratio (OR) with 95% confidence intervals (CIs) for hypertension across BMI trajectory categories.

The exclusion criteria were: (1) reporting self-anthropometric measures, such as weight and height, as exposure studies, (2) participants with diseases that may lead to the formation of secondary hypertension, (3) lack of diagnostic criteria for hypertension, (4) systematic review or meta-analysis, and (5) letters, meetings, or comments. Additionally, if duplicate studies originated from an identical or overlapping patient population, only the most recent and/or complete studies were used for the meta-analysis.

Titles and abstracts of the available studies were screened according to the inclusion and exclusion criteria after removing duplicates by blinded review. The full text of the remaining articles was independently examined to assess suitability.

According to the BMI classification standard organised by the World Health Organisation (8), there were five categories of BMI trajectories. The “Stable high” trajectory is characterised by BMI ≥ 25 kg/m2 and the range of change in BMI ≤ 1 kg/m2. The “Stable normal” trajectory is characterised by 18.5 ≤ BMI ≤ 24.9 kg/m2 and the range of change in BMI ≤ 1 kg/m2. The “Stable low” trajectory is characterised by BMI ≤ 18.5 kg/m2 and the range of change in BMI ≤ 1 kg/m2. The “Fluctuated (elevated-decrease)” trajectory features a trend of initially elevated and then decreased BMI. The difference between the highest and lowest BMI is ≤ 3 kg/m2. Finally, “Fluctuated (sharp-increase)” features the continued sharp increase of BMI >3 kg/m^2^.

### 2.2. Data extraction and assessment of study quality

Two reviewers (LT and L-zL) independently extracted the data from all eligible cohort studies. The characteristics extracted from each study included the last name of the first author and publication year; location of the study (country); study name; sex, sample size, number of individuals with high blood pressure; age range; length of follow-up; identification outcome method; baseline BMI, number of BMI measurements; adjustments.

Analyses of the extracted data included the number of individuals with hypertension and participants and reported RRs and 95% CIs in each trajectory. Alternatively, data to estimate RRs and CIs were used when a study did not report these parameters. For studies that did not report sufficient data to estimate RR and CI, we contacted the authors by email. Cases of disagreement were discussed and resolved by consensus by the two reviewers together with the participation of a third reviewer.

The Newcastle–Ottawa Scale (NOS) was used to assess the quality of cohort studies. The NOS was developed by Cochrane and is recommended to evaluate the potential biases associated with observational studies. According to the quality assessment criteria of the NOS (9), the overall bias of studies is based on three main factors: selection of cohort studies, comparability between groups, and identification of outcomes of interest.

Two reviewers (LT and HQ) assessed the risks of bias independently. When data were not reported in the main publications or supporting information, we contacted the corresponding author of the articles to provide them. Disagreements were resolved through consensus.

### 2.3. Data synthesis and statistical analyses

We performed a quantitative meta-analysis of relevant parameters based on Bayes’ theorem to establish a comprehensive estimate of the impact of different BMI trajectories on the risk of hypertension. This statistical method can combine the results of direct and indirect comparisons of multiple treatments while preserving the benefits of randomised comparisons within each trial.

All data analyses and image optimisation were performed using STATA version 15.1 (Stata Corp., College Station, TX, USA) and R version 4.1.2, using the “netmeta” and “gemtc” packages (10). To confirm the assumption of overall consistency between networks in the meta-analysis, the outcomes of the evaluation were statistically confirmed. Subsequently, for the local test of loop inconsistency, we used the separating indirect from direct evidence (SIDE) splitting method. When there were no inconsistencies in both the global and local inspections, a consistent model was accepted.

A network meta-analysis was performed using R software to compare the direct and indirect evidence of exposure factors in the included studies (11). The model used four Markov chains to set the initial value of 2.5. Twenty thousand pre-iterations were used for annealing. Fifty thousand iterations were used to achieve model convergence. The convergence of the trace and density maps was examined. When the potential scale reduction factor (PSRF) tends towards 1, the model convergence was deemed satisfactory; otherwise, the number of iterations continued to increase.

Relative risk and 95% CI were chosen as the effect sizes to report the results of the meta-analysis. Studies that reported effect estimates as HRs or ORs were considered equal to the RRs. We used the common logarithm transformations of RRs and their 95% CIs to estimate standard errors. The Higgins I-squared (*I*^2^) index was used to assess the potential heterogeneity. *I*^2^ degrees of 0, 25, 50, and 75% were considered thresholds for missing, low, moderate, and high heterogeneity, respectively. Moderate and above heterogeneity adopted a random-effects model.

In the presence of significant heterogeneity, meta-regression and subgroup analyses were performed to explore relevant sources of heterogeneity. Notably, the variables used in the meta-regression were limited to those with credible evidence of clinical or epidemiological effects based on a sufficient number of studies to ensure statistical power (4). Subgroup analyses were performed to assess the effects of age, sex, baseline BMI, follow-up time, sample size, study design, and trajectory modelling method on the pooled RR.

Forest and prediction interval plots were constructed to compare the effects of different BMI trajectories on the risk of developing hypertension. The ranking was performed using the surface under the cumulative ranking curve area (SUCRA) to determine the advantages of causing the onset of hypertension.

Sensitivity analyses were performed by pooling the studies with a low overall risk of bias to assess the robustness of the results. Specifically, the leave-1-out method was used to repeat the random-effects model by omitting one study at a time.

### 2.4. Grading evidence

We assessed the certainty of evidence using the GRADE system. Network estimates of observational studies and the direct and indirect estimates that make up these network estimates began with low certainty of evidence. These were downgraded by established criteria for risk of bias, inconsistency (incoherence), indirectness, imprecision, and publication bias. The certainty of the evidence was upgraded by large effect sizes, dose-response relationships, and residual confounding. The evidence grading standards were “very low,” “low,” “moderate,” and “high.” A higher rating indicated that the estimated effect was closer to the true effect size.

## 3. Results

### 3.1. Study selection

**FIGURE 1:**
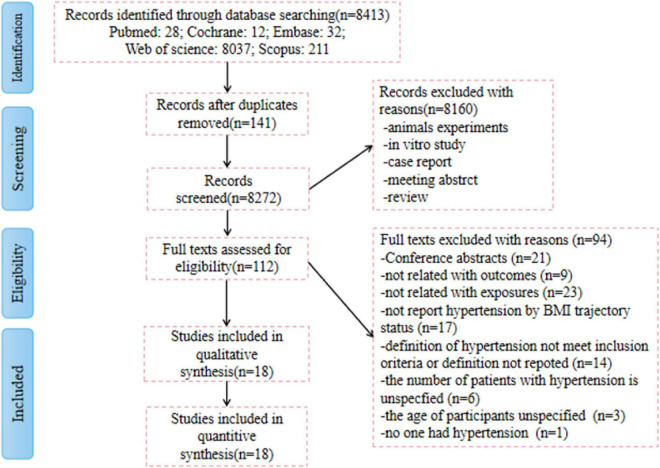
Preferred Reporting Items for Systematic Reviews and Meta-Analyses (PRISMA) flow diagram of the study selection process.

A total of 8,413 records were identified in the initial search of the databases and reference lists. After excluding 141 duplicates and 8,160 unrelated articles based on screening of the title and abstract, 112 full-text publications were reviewed for eligibility. Ultimately, 18 articles provided sufficient information and were considered qualified for our meta-analysis (6, 12–28). The 18 cohort studies included 89,094 participants. The patients included 30,146 with hypertension, accounting for 33.83% of the total. All included studies were original and published between 2015 and 2021. The studies were cohort studies conducted in the general population of any age group. Patients with a medical history of hypertension at baseline were excluded. The network of included studies is shown in Figure 1.

### 3.2. Study characteristics

A comprehensive analysis of the selected studies is shown in Table 1. Sample sizes ranged from 664 to 28,706. Participants ranged in age from newborn to 101 years of age, with a median age of 42.5 years. All studies included a range of geographical areas in Asia (*n* = 14), North America (*n* = 2), Australia (*n* = 1), and South Africa (*n* = 1). In terms of trajectory determination, the number of repeat measurements of BMI ranged from 3 to 22 during follow-up. Ten studies used the latent class growth mixture model (LCGMM) trajectory modelling approach, five studies used latent class growth analysis (LCGA), two studies used group-based trajectory models (GBTM), and one study used latent class trajectory (LCTA). Six articles had a retrospective cohort design and 12 articles had a prospective cohort design.

**TABLE 1:** Overview of included publications.

First author (Publication year)	Country/ Region	Study name	Gender	Participants/ Cases	Age range (mean), years	Follow-up (years)	Outcome identification method	Number of measure ments of BMI	Trajectory modelling method	baseline BMI (kg/m^2^)
Ji et al. (12)	China	China Health and Nutrition Survey	W/M	1,907/179	5–19 (8.6)	7.3	SBP/DBP ≥140/90 mmHg or medical records or taking antihypertensive medication	3∼6	LCGMM	23.5
Wang et al. (13)	China	the National Natural Science Foundation of China	W/M	9,286/1,569	7–18 (8.2)	9	BP greater than or equal to the 95th percentile for children younger than 13 years, and BP greater than or equal to 130/80 mmHg for children aged 13 years or older.	8.45	GBTM	17.8
Teng et al. (14)	China	China Health and Nutrition Survey	W/M	1,872/162	6–18 (11.52)	19	Self-reported hypertension, SBP/DBP ≥140/90 mmHg or using antihypertension medications	4.11	LCGMM	17.68
Fan and Zhang (15)	China	China Health and Nutrition Survey	W/M	1,484/409	3–13 (8.1)	6.5	BP ≥120/80 mmHg or 90th percentile by sex, age, and height (whichever is lower) as per China’s national BP reference for children.	3∼5	GBTM	16.64
Fan et al. (16)	China	the National Natural Science Foundation of China	W/M	3,271/385	20–40 (28.7)	5.5	SBP/DBP ≥140/90 mmHg or taking antihypertensive medication or diagnosis by medical records.	4∼11	LCGMM	23.93
Zeng et al. (17)	China	China Health and Nutrition Survey	W/M	4,697/1,954	40–70 (43)	11.51	SBP/DBP ≥140/90 mmHg or through self-report	4.48	LCGMM	22.23
Xu et al. (18)	China	National Key Research and Development Program of China	W/M	3,939/1,357	35–82 (45.9)	11.24	SBP/DBP ≥140/90 mmHg or use of BP lowering medications within 2 weeks	3	LCGMM	22.22
Liu et al. (19)	China	China Health and Nutrition Survey	W/M	1,4262/5,138	24–50 (38.8)	9.6	SBP/DBP ≥140/90 mmHg or the use of antihypertensive medications, or a physician diagnosis of hypertension	9	LCTA	21.15
Guo et al. (20)	China	National Key Research and Development Program of China	W/M	28,706/14,403	50–101 (64.24)	8	SBP/DBP ≥140/90 mmHg or with a previous history of hypertension or taking antihypertensive drugs	8	LCGA	23.19
Islam et al. (21)	Japan	China Health and Nutrition Survey	W/M	5,276/784	6–80 (34.8)	20	SBP/DBP ≥140/90 mmHg or self-report of currently using antihypertensive medication	7	LCGA	23.34
Buscot et al. (22)	Australia	Competitive State Research	W/M	2,631/613	6–18 (12.11)	31	SBP/DBP ≥140/90 mmHg or higher, or taking antihypertensive drugs	5.4	LCGMM	18
Yuan et al. (23)	China	The Hanzhong Adolescent Hypertension Cohort Study	W/M	2,839/250	6–18 (11.52)	30	SBP/DBP ≥140/90 mmHg or current use of antihypertensive medications	3∼6	LCGMM	17.67
Ahanchi et al. (6)	Japan	The Tehran Lipid and Glucose Study	W/M	664/111	12–20 (15.46)	12	SBP/DBP ≥140/90 mmHg or using anti-hypertension medications.	5	LCGMM	24.37
Aris et al. (24)	Singapore	Singapore Clinical Sciences Research	W/M	1,170/757	0–2 (1)	5	SBP or DBP above the 90th percentile for the child’s sex, age and height.	9	LCGMM	25.1
Wang et al. (25)	Canada	National Population Health Survey	W/M	3,070/713	40–70 (43)	15	SBP/DBP ≥140/90 mmHg or using antihypertension medications	4	LCGA	26.55
Munthali et al. (26)	South Africa	the South African Medical Research	W/M	1,824/552	5∼18 (11.5)	13	SBP/DBP ≥140/90 mmHg	13	LCGMM	17.5
Naseri et al. (27)	Iran	Endocrine Sciences research	W	1,356/318	51–55 (51.43)	20	SBP/DBP ≥140/90 mmHg or taking antihypertensive drugs	5	LCGA	30.48
Ford et al. (28)	USA	Institute of Nutrition for Central America and Panama	W/M	856/492	37∼54 (46.0)	40	SBP/DBP ≥130/80 mmHg or anti-hypertensive medication use	22	LCGA	28.61

W/M, women/men; SBP, systolic blood pressure; DBP, diastolic blood pressure; LCGMM, latent class growth mixture model; GBTM: group-based trajectory modelling; LCGA, latent class growth analysis; LCTA, latent class trajectory analysis.

All studies reported multivariable effect estimates. There were 35 types of variables. Among them, 18 studies reported controlling for age, 12 studies for gender and smoking status, eight studies for alcohol consumption and education level, seven studies for systolic blood pressure and socioeconomic status, six for living conditions, and five for ethnicity. BMI, working status, marital status, diastolic blood pressure, gestational age, and birth weight were confounding factors and were controlled for in four studies each. Finally, one to three studies were conducted to control for the following confounders: diabetes history, hyperlipidaemia history, hypertension history, cardiovascular disease history, pulse wave velocity, survey wave, physical activity, and dietary energy intake, pregnancy BMI, paternal BMI, puberty status, breastfeeding duration, hypertension in pregnancy, gestational diabetes, family adversity index, birth village, gestational weight gain, and parity.

### 3.3. Results of traditional meta-analysis

The hypertension risk was not entirely consistent across the BMI trajectory categories in the traditional meta-analysis. Compared with the “Stable normal” group, the “Stable high,” “Fluctuated (elevated-decrease),” and “Fluctuated (sharp-increase)” trajectories each had a higher morbidity risk, while the “Stable low” trajectory had a lower risk of hypertension.

#### 3.3.1. “Stable high”

Five original studies documented the association of the “Stable high” trajectory with the onset of hypertension. Of the total of 10,073 participants, 4,271 ultimately developed hypertension, representing an incidence rate of 42.40%. The “Stable high” BMI trajectory displayed an 80% increased risk of hypertension compared to the “Stable normal” trajectory [RR = 1.80, 95% CI (1.29, 2.50), *I*^2^ = 98.4%, *p* < 0.001] (Supplementary Figure 1A).

#### 3.3.2. “Stable low”

Three studies mentioned the relationship between the “Stable low” trajectory and the occurrence of hypertension. Of the total of 5,012 participants, 912 developed high blood pressure, representing an incidence rate of 18.20%. The meta-analysis of the fixed-effects model showed that the “Stable low” trajectory was associated with a lower risk of developing hypertension than the “Stable normal” trajectory. [RR = 0.83, 95% CI (0.79, 0.87), *I*^2^ = 0.0%, *p* = 0.994] (Supplementary Figure 1B).

#### 3.3.3. “Fluctuated (elevated-decrease)”

Eight studies investigated the association of the “Fluctuated (elevated-decrease)” trajectory with the risk of developing hypertension. Of the total of 7,907 participants, 2,981 people ultimately developed hypertension, representing an incidence rate of 37.70%. The “Fluctuated (elevated-decrease)” trajectory was associated with a 30% increased risk of hypertension compared to the “Stable normal” trajectory [RR = 1.30, 95% CI (1.24, 1.37), *I*^2^ = 13.8%, *p* = 0.322] (Supplementary Figure 1C).

#### 3.3.4. “Fluctuated (sharp-increase)”

The association of this trajectory with the risk of developing hypertension was reported in 16 studies. Of the total of 29,861 patients, 10,703 ultimately developed hypertension, representing an incidence rate of 35.8%. The “Fluctuated (sharp-increase)” trajectory was associated with a 53% increased risk of hypertension [RR = 1.53, 95% CI (1.27, 1.83), *I*^2^ = 86.8%, *p* < 0.001] (Supplementary Figure 1D).

### 3.4. Results of network meta-analysis

Supplementary Figure 2 showed the direct and indirect comparisons between the five categories of trajectories, presented as a visual network evidence map. The map included the study population for the five categories of BMI trajectory frequency and the most accurate direct comparison. Seven closed loops were formed. The number of direct comparison studies on the “Fluctuated (sharp-increase)” trajectory and the “Stable normal” trajectory was the largest.

In the node split models, all comparison results in the table were not significantly different (*p* > 0.05) (Table 2). The findings indicated the good consistency between the direct and indirect comparisons of the included studies and further indicated that the data analysis should be performed using the consistency model.

**TABLE 2:** Evaluation of local inconsistency for direct and indirect comparisons using node split model.

Name	Direct effect		Indirect effect		Overall		*P*-value
A, B	0.0284837	0.3373684	−1.089848	0.6795004	1.118332	0.7480262	0.135
A, D	0.4825778	0.820253	0.7333531	0.4466837	−0.2507753	0.9393694	0.789
A, E	0.3707947	0.3717866	1.331222	0.6880083	−0.9604271	0.8064972	0.234
B, C	0.2928055	0.2473767	0.0869463	0.5882546	0.2058592	0.6164788	0.738
B, D	0.8249175	0.2782698	1.092155	0.6619053	−0.2672375	0.7070956	0.705
B, E	0.7380913	0.1545764	2.576586	0.9649444	−1.838495	0.9739088	0.059
C, D	0.074921	0.5618024	0.8344288	0.3822245	−0.7595078	0.6794839	0.264
C, E	0.613181	0.2640012	0.0382403	0.5907842	0.5749408	0.6375694	0.367
D, E	0.0158358	0.3953693	−0.1608747	0.4165963	0.1767105	0.5835465	0.762

A, Stable low; B, Stable normal; C, Fluctuated (elevated-decrease); D, Stable high; E, Fluctuated (sharp increase).

Trace and density maps established using the Bayesian model are depicted in Supplementary Figure 3A. Each Markov Chain Monte Carlo chain achieved stable fusion from the beginning. The overlapping area accounted for most of the chain fluctuation range in the subsequent calculation. In addition, in the density map, a normal curve distribution was evident. The bandwidth of 0.01927 was close to the pre-set distribution. The findings indicate that the convergence degree of the model under *n*. adapt = 20,000 and *n*. inter = 50,000 is satisfactory.

In the Brooks–Gelman–Rubin diagnostic plot, the median and 97.5% reduction factors tended to be 1 and became stable after iterations. The PSRF also tended towards 1 (Supplementary Figure 3B). Therefore, the model converged satisfactorily, the total number of iterations was sufficient, and the results were relatively stable.

Supplementary Figure 4 shows a cumulative ranking plot drawn according to the likelihood that the five BMI trajectories lead to the occurrence of hypertension. In Table 3, the BMI trajectories of “Stable high” (SUCRA = 88.1%) had the highest probability of developing hypertension, immediately followed by another equally remarkable “Fluctuated (sharp-increase)” trajectory (SUCRA = 83.9%). SUCRA values of “Fluctuated (elevated-decrease),” “Stable normal” and “Stable low” trajectories were 37.6, 30.5, and 10.0%, respectively.

**TABLE 3:** Surface under the cumulative ranking curve area (SUCRA) values for various trajectories.

Exposure	SUCRA (%)	PrBest	Mean rank
Stable low	30.5	1.5	3.8
Stable normal	10	0	4.6
Fluctuated (elevated-decrease)	37.6	0.4	3.5
Stable high	88.1	59.2	1.5
Fluctuated (sharp-increase)	83.9	38.9	1.6

Concerning hypertension, Supplementary Figure 5 displays the following data. The “Stable high” trajectory was higher than that of “Fluctuated (sharp-increase)” [RR = 1.08, 95% CI (0.62, 1.85)]. The “Fluctuated (sharp-increase)” trajectory was higher than that of “Fluctuated (elevated-decrease)” [RR = 1.69, 95% CI (1.05, 2.72)]. The “Fluctuated (sharp-increase)” trajectory was higher than that of “Stable normal” [RR = 2.20, 95% CI (1.60, 3.03)]. The “Fluctuated (sharp-increase)” trajectory was higher than that of the “Stable low” [RR = 1.83, 95% CI (0.98, 3.41)]. The “Stable high” trajectory was higher than that of “Fluctuated (elevated-decrease)” [RR = 1.81, 95% CI (0.97, 3.39)]. The “Stable high” trajectory was higher than that of “Stable normal” [RR = 2.37, 95% CI (1.44, 3.89)]. The “Stable high” trajectory was higher than that of the “Stable low” [RR = 1.97, 95% CI (0.93, 4.17)]. The “Fluctuated (elevated-decrease)” trajectory was higher than that of “Stable normal” [RR = 1.31, 95% CI (0.83, 2.05)]. The “Fluctuated (elevated-decrease)” trajectory was higher than that of “Stable low” [RR = 1.09, 95% CI (0.52, 2.26)]. Finally, the “Stable normal” trajectory was lower than that of the “Stable low” [RR = 0.83, 95% CI (0.45, 1.53)].

### 3.5. Meta-regression analysis

Age (*p* = 0.017) and baseline BMI (*p* = 0.045) were identified as potential sources of heterogeneities between studies on the “Fluctuated (sharp increase)” trajectory by meta-regression analysis. The full details of the meta-regression are provided in Table 4 and Supplementary Figure 6.

**TABLE 4:** Results of meta-regression analysis of “Fluctuated (sharp-increase)” trajectory.

Relative risk	Exp (b)	Standard error	*t*	*P* > *t*	CI
Age	1.44924	0.199	2.70	**0.017**	1.079∼1.946
Follow-up time	1.502239	0.321	1.90	0.079	0.947∼2.383
Sample size	1.184251	0.244	0.82	0.426	0.759∼1.847
Region	1.533859	0.566	1.16	0.269	0.687∼3.426
Hypertension definition	1.957625	0.801	1.64	0.129	0.795∼4.818
Number of measurements of BMI	0.9668908	0.016	−2.02	0.063	0.933∼1.002
Baseline BMI	0.951519	0.021	−2.20	**0.045**	0.907∼0.999
Trajectory modelling method	1.041228	0.259	0.16	0.874	0.605∼1.792
Study design	1.015463	0.203	0.08	0.940	0.661∼1.559
Age adjustment	0.7024152	0.129	−1.92	0.075	0.474∼1.042
Gender adjustment	1.217845	0.224	1.07	0.302	0.821∼1.807
SBP adjustment	0.8126039	0.151	−1.12	0.283	0.546∼1.120
Living condition adjustment	0.8189415	0.166	−0.99	0.340	0.531∼1.264
Smoking adjustment	0.7750825	0.132	−1.50	0.157	0.538∼1.117
Drinking adjustment	0.9872743	0.189	−0.07	0.948	0.655∼1.487
Physical activity adjustment	0.8037896	0.156	−1.12	0.280	0.530∼1.220
Education level adjustment	1.130922	0.241	0.58	0.572	0.716∼1.785
BMI adjustment	0.7909991	0.149	−1.24	0.234	0.528∼1.186
Marital status adjustment	1.0487	0.237	0.21	0.836	0.646∼1.702

Bold values mean P < 0.05, indicating a significant regression relationship between the two variables.

### 3.6. Subgroup analysis

Heterogeneities between studies for “Fluctuated (sharp increase),” “Stable high,” and “Fluctuated (elevated-decrease)” trajectories were separately identified by subgroup analysis (Table 5 and Supplementary Figures 7–9).

**TABLE 5:** Relationships between different body mass index (BMI) trajectories and risk of hypertension based on subgroup analysis.

BMI trajectory	Fluctuated (sharp-increase)					Fluctuated (elevated-decrease)					Stable high				
**Subgroup**	**No. of studies**	**RR (95% CI)**	***P*-value**	***I*^2^ (%)**	**P (heterog eneity)**	**No. of studies**	**RR (95% CI)**	***P*-value**	***I*^2^ (%)**	**P (heterog eneity)**	**No. of studies**	**RR (95% CI)**	***P*-value**	***I*^2^ (%)**	***P* (heterog eneity)**
Age, years															
<20	9	1.716 (1.573∼1.873)	0.000	0.0	0.896	4	0.926 (0.592∼1.449)	0.736	1.3	0.386	1	1.230 (0.469∼3.228)	0.674	–	–
>20	7	1.272 (1.057∼1.531)	0.011	79.2	0.000	4	1.308 (1.245∼1.374)	0.000	0.0	0.419	4	1.843 (1.155∼2.940)	0.000	98.8	0.000
Gender															
Male	10	1.608 (1.373∼1.883)	0.000	45.9	0.055	6	1.301 (1.238∼1.367)	0.000	5.5	0.381	3	1.783 (0.970∼3.277)	0.063	99.2	0.000
Female	6	1.329 (1.007∼1.755)	0.045	82.4	0.000	2	1.372 (0.961∼1.960)	0.082	63.5	0.098	2	1.776 (1.321∼2.387)	0.000	88.1	0.004
Region															
Asia	13	1.599 (1.298∼1.971)	0.000	88.0	0.000	6	1.305 (1.242∼1.371)	0.000	20.7	0.278					
America	1	0.852 (0.622∼1.167)	0.317	–	–										
Oceania	1	1.810 (1.356∼2.415)	0.000	–	–	1	0.760 (−0.347∼1.666)	0.493	–	–					
Africa	1	1.534 (0.909∼2.590)	0.109	–	–	1	1.340 (0.550∼3.263)	0.519	–	–					
Hypertension definition															
SBP/DBP ≥140/90 mmHg, or medical records, or antihypertensive medication	4	1.407 (0.991∼1.998)	0.056	73.2	0.011	2	0.999 (0.526∼1.898)	0.999	79.9	0.026	1	1.381 (1.300∼1.467)	0.000	–	–
SBP/DBP ≥140/90 mmHg, or antihypertensive medication	5	1.971 (1.340∼2.897)	0.001	74.4	0.004	4	1.358 (1.050∼1.756)	0.020	0.0	0.401	2	2.376 (1.757∼3.214)	0.000	97.7	0.000
SBP/DBP ≥140/90 mmHg	3	1.353 (1.106∼1.654)	0.003	0.0	0.871	1	1.340 (0.550∼3.263)	0.519	–	–	2	1.492 (1.233∼1.806)	0.000	0.0	0.689
SBP/DBP ≥130/80 mmHg	2	1.218 (0.625∼2.375)	0.562	93.9	0.000										
SBP/DBP ≥120/80 mmHg	2	1.912 (1.066∼3.430)	0.030	0.0	0.726	1	1.580 (0.493∼5.065)	0.442	–	–					
Sample size															
<5,000	12	1.511 (1.250∼1.826)	0.000	55.0	0.011	6	1.092 (0.834∼1.430)	0.521	8.3	0.363	4	2.000 (1.522∼2.628)	0.000	95.4	0.000
5,000∼10,000	2	2.280 (1.031∼5.043)	0.042	71.0	0.063	1	1.600 (1.073∼2.387)	0.021	–	–					
>10,000	2	1.159 (0.868∼1.548)	0.317	67.6	0.079	1	1.306 (1.242∼1.373)	0.000	–	–	1	1.381 (1.300∼1.467)	0.000	–	–
Follow-up time, years															
≤10	7	1.529 (1.129∼2.069)	0.006	92.7	0.000	3	1.300 (1.237∼1.367)	0.000	60.6	0.079	2	1.380 (1.300∼1.466)	0.000	0.0	0.815
10∼20	6	1.446 (1.164∼1.797)	0.001	40.0	0.139	4	1.446 (1.114∼1.876)	0.006	0.0	0.892	3	2.068 (1.561∼2.741)	0.000	96.8	0.000
> 20	3	1.548 (0.827∼2.899)	0.172	88.4	0.000	1	0.760 (−0.347∼1.666)	0.493	–	–					
Trajectory modelling method															
LCGMM	10	1.425 (1.286∼1.580)	0.000	36.6	0.116	5	1.070 (0.811∼1.411)	0.633	20.8	0.282	2	1.492 (1.233∼1.806)	0.000	0.0	0.689
GBTM	2	1.688 (1.535∼1.857)	0.000	0.0	0.768	1	1.580 (0.493∼5.065)	0.442	–	–					
LCGA	3	1.169 (0.935∼1.463)	0.171	83.7	0.002	2	1.310 (1.246∼1.377)	0.000	0.0	0.324	3	1.947 (1.877∼2.020)	0.000	99.2	0.000
LCTA	1	1.208 (1.159∼1.260)	0.139	–	–										
Study design															
Prospective cohort study	10	1.518 (1.259∼1.831)	0.000	70.5	0.000	4	1.304 (1.241∼1.370)	0.000	0.0	0.607	3	1.623 (1.135∼2.321)	0.008	97.3	0.000
Retrospective cohort study	6	1.528 (1.273∼1.835)	0.009	74.4	0.002	4	1.245 (0.907∼1.708)	0.175	51.6	0.102	2	2.057 (1.130∼3.744)	0.018	97.1	0.000
Baseline BMI															
BMI < 18.5 kg/m^2^	6	1.716 (1.572∼1.874)	0.000	0.0	0.653	2	0.974 (0.541∼1.755)	0.930	0.0	0.349	1	1.230 (0.469∼3.222)	0.674	–	–
18.5 < BMI ≤ 24.9 kg/m^2^	8	1.386 (1.140∼1.684)	0.001	75.5	0.000	6	1.305 (1.242∼1.371)	0.000	20.7	0.278	3	1.796 (1.059∼3.048)	0.030	99.2	0.000
25 ≤ BMI ≤ 30 kg/m^2^	2	0.868 (0.637∼1.183)	0.371	0.0	0.518										
BMI > 30 kg/m^2^											1	2.037 (1.909∼2.174)	0.000	–	–

#### 3.6.1. “Stable high” trajectory

Data from five studies were used to assess the risk of hypertension in the “Stable high” trajectory compared with the “Stable normal” trajectory. The “Stable high” trajectory significantly increased the risk of hypertension among the two trajectory modelling methods of LCGMM (RR = 1.49; 95% CI:1.23∼1.81) and LCGA (RR = 1.95; 95% CI:1.88∼2.02). Compared with the “Stable normal” trajectory, the “Stable high” trajectory had a higher risk of hypertension for both subgroups with follow-up times <10 years (RR = 1.38; 95% CI:1.30∼1.47) and 10–20 years (RR = 2.07; 95% CI:1.56∼2.74).

#### 3.6.2. “Fluctuated (elevated-decrease)” trajectory

The risk of hypertension was compared between the “Fluctuated (elevated-decrease)” and “Stable normal” trajectories based on data from eight studies. The “Fluctuated (elevated-decrease)” trajectory had a significantly higher risk of hypertension for adults >20-years-of-age (RR = 1.31; 95% CI:1.24∼1.37), while the hypertension risk was not significantly higher for children within the age of 20 years (RR = 0.93; 95% CI:0.59∼1.45).

#### 3.6.3. “Fluctuated (sharp-increase)” trajectory

Comparison of the “Fluctuated (sharp-increase)” and “Stable normal” trajectories of the reference group revealed significant differences in heterogeneities between strata by age and baseline BMI based on the data from 16 studies. The findings were consistent with those of the meta-regression analysis. In the subgroup analysis, compared with the “Stable normal” trajectory, the “Fluctuated (sharp-increase)” trajectory displayed an increased incidence of hypertension in both children (RR = 1.72; 95% CI:1.57∼1.87) and adults (RR = 1.27; 95% CI:1.06∼1.53). Compared with the “Stable normal” trajectory, the “Fluctuated (sharp-increase)” trajectory displayed a significantly higher risk of hypertension among the groups of baseline BMI <18.5 kg/m^2^ (*R* = 1.72; 95% CI:1.57∼1.87) and 18.5 ≤ baseline BMI ≤ 24.9 kg/m^2^ (RR = 1.39; 95% CI:1.14∼1.68). The “Fluctuated (sharp-increase)” trajectory was not significantly higher in hypertension risk compared to the “Stable normal” trajectory for 25 ≤ baseline BMI < 30 kg/m^2^ (RR = 0.87; 95% CI:0.64∼1.18).

### 3.7. Sensitivity analysis

Leave-1-out method of sensitivity analysis results indicated that the exclusion of any single study did not affect the results of the overall meta-analysis of the various BMI trajectories. The findings suggest that the overall meta-analysis results are robust concerning the potential effects of bias.

### 3.8. Publication bias assessment

As shown in Supplementary Figure 10, we created a comparison-adjusted funnel plot with the effect size of each indicator as the abscissa and standard error as the ordinate. Each dot represents a direct comparison of different interventions. The number of dots in the same colour represents the number of pairwise comparisons in this study. The comparison-adjusted funnel plot showed that all studies were distributed on both sides of the midline in the middle, and the left and right distributions were roughly symmetrical. However, several small studies were distributed outside the inverted funnel, suggesting a possible publication bias. Careful analysis of the data revealed that the bias was not caused by clinical methods or statistical problems. These articles all met the literature inclusion criteria and should not be excluded. Therefore, inclusion and statistical analyses should continue.

### 3.9. Quality of included study

As shown in Table 6, high scores were reported for all assessment trials, ranging from a minimum of 7 to a maximum of 9, based on the NOS criteria. Controlling for age as the most important variable, cohort comparability achieved the highest score. A qualitative assessment of the included studies showed that their overall quality was high.

**TABLE 6:** Newcastle-Ottawa Score (NOS) calculation for studies included in the systematic review.

Study	Selection				Comparability	Outcome			NOS Score
	**Representativeness of the exposed cohort**	**Selection of the non-exposed cohort**	**Ascertainment of exposure**	**Outcome of interest not present at the start of the study**	**Comparability of controls**	**Outcome assessment**	**Sufficient follow-up duration**	**Adequacy of follow-up**	**Overall bias**
Ji et al. (12)	Y	Y	Y	Y	Y	Y	N	Y	8
Wang et al. (13)	Y	Y	Y	Y	Y	Y	N	N	7
Teng et al. (14)	Y	Y	Y	Y	Y	Y	Y	N	8
Fan and Zhang (15)	Y	Y	Y	Y	Y	Y	N	Y	8
Fan et al. (16)	Y	Y	Y	Y	Y	Y	N	Y	8
Zeng et al. (17)	Y	Y	Y	Y	Y	Y	Y	N	8
Xu et al. (18)	Y	Y	Y	Y	Y	Y	Y	Y	9
Liu et al. (19)	Y	Y	Y	Y	N	Y	Y	Y	7
Guo et al. (20)	Y	Y	Y	Y	Y	Y	Y	N	8
Islam et al. (21)	Y	Y	Y	Y	Y	N	Y	N	7
Buscot et al. (22)	Y	Y	Y	Y	Y	Y	Y	Y	9
Qu et al. (29)	Y	Y	Y	Y	Y	N	Y	N	7
Ahanchi et al. (6)	Y	Y	Y	Y	Y	Y	Y	N	8
Aris et al. (24)	Y	Y	Y	Y	Y	N	N	Y	7
Wang et al. (25)	Y	Y	Y	Y	Y	N	Y	Y	8
Munthali et al. (26)	Y	Y	Y	Y	Y	Y	Y	N	8
Naseri et al. (27)	Y	Y	Y	Y	Y	N	Y	Y	8
Ford et al. (28)	Y	Y	Y	Y	Y	N	Y	N	7
								

### 3.10. GRADE assessment

Supplementary Table 4 depicts the GRADE assessment data for the network meta-analysis. The certainty of the evidence for direct comparisons of the incidence of hypertension between various BMI trajectories was generally very low. In five of the nine direct comparisons, degradation was due to indirectness and imprecision: “Stable low” vs. “Stable normal” trajectories, “Stable low” vs. “Stable high” trajectories, “Fluctuated (sharp-increase)” vs. “Stable low” trajectories, “Fluctuated (elevated-decrease)” vs. “Stable normal” trajectories, and “Stable high” vs. “Fluctuated (sharp-increase)” trajectories. In addition, the comparison between the degradation of the “Stable high” and “Stable Normal” trajectories revealed the influences of indirectness and imprecision, as well as heterogeneity. The degradation of the “Fluctuated (sharp-increase)” trajectory vs. the “Stable normal” trajectory, and the “Fluctuated (sharp-increase)” trajectory vs. “Fluctuated (elevated-decrease)” trajectory reflected indirection and heterogeneity. Comparison of the degradation between the “Stable high” and “Fluctuated (elevated-decrease)” trajectories revealed the influence of indirectness alone.

## 4. Discussion

To the best of our knowledge, this is the first global systematic review and meta-analysis to assess BMI trajectories in relation to the risk of developing hypertension. Compared with the “Stable normal” trajectory, the “Stable high” trajectory had the highest increased risk of hypertension. The “Fluctuated (sharp-increase)” trajectory and the “Fluctuated (elevated-decrease)” trajectory were also associated with an increased risk of hypertension. The “Stable low” trajectory displayed a lower risk of hypertension. A high degree of heterogeneity was evident for the “Stable high” and “Fluctuated (sharp-increase)” trajectories. Subgroup analyses revealed a consistent and positive association between “Stable high” and hypertension risk, regardless of the trajectory modelling method and follow-up time. Meta-regression and subgroup analyses consistently implicated age and baseline BMI as pivotal factors contributing to the significant heterogeneity among studies in the “Fluctuated (sharp-increase)” trajectory.

A 2017 meta-analysis showed that BMI ≥30 kg/m^2^ at a single point in time was not a significant predictor of hypertension, as it may mask the effect of age. Subsequently, scholars began to pay attention to the correlation between the dynamic change in BMI and hypertension and confirmed a non-linear dose-response relationship between the continuous change in BMI and hypertension (29). Another dose-response meta-analysis found that the incidence of hypertension was linearly associated with an increase in BMI (4). However, no study has established a relationship between different continuous trends of BMIs and hypertension risk. Unlike the former studies, the present analysis identified a relationship between different BMI trajectories and hypertension risk. The results by age were consistent with data from systematic reviews of different age groups. For example, Friedemann et al. (30) performed a systematic review of blood pressure in overweight and obese healthy children and found that elevated BMI was associated with higher systolic, diastolic, and 24-h ambulatory systolic blood pressures. Similarly, Piper et al. (31) performed an updated systematic review of the benefits and harms of adult hypertension screening and found that overweight and obese adults with increased BMIs had nearly twice the incidence of hypertension in people with normal BMIs.

Our findings provide new insights into the development of hypertension and have significant policy implications for its management. The findings highlight that continuous BMI monitoring is essential to identify the onset of early hypertension. Especially in populations with rapid BMI gain or high BMI values, the global health system should formulate specific public policies and clinical considerations, and implement more effective public health interventions to prevent hypertension at an early stage.

A cross-sectional study of 5,171 participants showed that younger individuals were more likely to develop hypertension than older adults in the same state of persistently increasing BMI (32). These findings suggest that different age groups have different pathophysiological responses to the “Fluctuated (sharp-increase)” trajectory. Consistent with this explanation, our meta-analysis suggested that compared with the “Stable normal” trajectory, the “Fluctuated (sharp-increase)” trajectory increased the hypertension risk in children and adolescents (RR = 1.72; 95% CI:1.57∼1.87). Furthermore, the increased risk exceeded that in adults (RR = 1.27; 95% CI:1.06∼1.53). Zhou et al. performed a dose-response meta-analysis (4) that revealed a greater risk of hypertension in a population with a baseline BMI ≤25 kg/m^2^. The RR of hypertension increased by increments of five 1.50 units in BMI. Our findings on the risk of hypertension are consistent with those of the prior meta-analysis. Since the baseline BMI of children is usually <25 kg/m^2^, this also partly explains the higher risk of hypertension in children compared to adults.

Current research on whether the “Fluctuated (elevated-decrease)” trajectory is associated with the risk of hypertension remains controversial. Buscot et al. (22) described that compared with the “Stable Normal” trajectory, the “Fluctuated (elevated-decrease)” trajectory had a reduced risk of hypertension (HR = 0.76). However, Munthali et al. (26) described that the “Fluctuated (elevated-decrease)” trajectory was associated with an increased risk of developing hypertension [RR = 1.34, (95% CI:0.72 to 2.50)] compared with the “Stable normal” trajectory. Our findings were consistent with those of Munthali et al. Furthermore, our subgroup analysis showed that children and adults are not homogeneous groups in terms of “Fluctuated (elevated-decrease)” trajectory and hypertension risk. Compared with the “Stable normal” trajectory, the “Fluctuated (elevated-decrease)” trajectory without an increase in hypertension risk for children. In contrast, the risk of hypertension was significantly increased in adults.

Our results suggest that clinicians and policymakers should adopt tailored BMI management strategies for different age groups. In children and adolescents, a rapid increase in BMI is a crucial indicator of the onset of hypertension, and children who revert their overweight/obesity to a normal BMI may have a reduced risk of hypertension. Thus, continuous BMI monitoring is essential for the early identification of high-risk children and timely management of risk factors related to hypertension, such as autonomic dysfunction, insulin resistance, and abnormal vascular structure and function, which are often accompanied by childhood overweight/obesity. In adults, a sharp increase in BMI or a stable high BMI are significant risk factors for the onset of hypertension. In addition, the unstable fluctuation of BMI from overweight to normal, or from below normal to normal was still associated with a higher risk of hypertension compared to a stable normal BMI. Therefore, unstable BMI may be a strong determinant of the risk of adult hypertension. Maintaining a Stable normal BMI in adults will likely be one of the effective measures to lower the incidence of hypertension in society. In addition, the implementation of more effective public health interventions may help to prevent hypertension at an early stage in adults with rapid BMI gain or high BMI.

The key to controlling BMI at a Stable normal level is a better understanding of what causes hypertension across the three types of BMI trajectories that contribute to hypertension, including “Stable high,” “Fluctuated (sharp-increase),” and “Fluctuated (elevated-decrease)” trajectories. There are several potential explanations for this, as discussed next.

The “Stable high” trajectory means that BMI is consistently maintained at a high level. Several pathophysiological mechanisms could potentially explain the association between stable high BMI levels and the development of hypertension. These mechanisms include lipid disorders (33), chronic low-grade inflammation (34), activation of the renin–angiotensin–aldosterone system (RAAS) (35), overdrive of sympathetic nerves (36), insulin resistance (IR) (37, 38), and gut microbiota dysbiosis (39, 40). Specifically, high BMI levels lead to adipose tissue dysfunction, increased adipocyte volume, decreased adiponectin secretion, and increased monocyte chemoattractant protein-1 (MCP-1) expression level. These alterations in turn recruit inflammatory immune cells, such as macrophages, to infiltrate and accumulate. As well, activated immune cells further release inflammatory cytokines, such as tumour necrosis factor-alpha (TNF-α), interleukin (IL)-1, and IL-6, which allow more monocytes to infiltrate the adipose tissue to form a systemic chronic low-grade inflammation. Due to the cumulative effect of adipose tissue in a stable high BMI state, the secretion of free fatty acids (FFAs) is also significantly increased. FFAs mediate macrophage metabolic disorders by forming small lipid mediators, including changes in circulating glucose and fatty acid substrates, lipidic toxicity, and tissue hypoxia, and induces M1 macrophage polarisation, thereby disrupting M1/M2 homeostasis and exacerbating inflammatory responses. In addition, both FFAs and inflammatory factors mediate IR by activating multiple inflammatory signalling pathways. For example, FFAs promote IR by activating the innate immune receptor Toll-like receptor 4 pathway. Inflammatory factors can activate the amino-terminal kinase/stress-activated protein kinase (JNK/SAPK) pathway to inhibit the insulin signalling pathway, resulting in IR. IR further enhances the excitability of the sympathetic nervous system, increases the reabsorption of water and sodium by the kidneys, causes the body to retain water and sodium, increases the sensitivity of blood pressure to salt, and induces vascular smooth muscle cell proliferation. Together, these factors contribute to the outcomes of hypertension. In addition, adipose tissue can also produce angiotensinogen, especially intra-abdominal adipocytes, which can produce angiotensin II and aldosterone, thereby activating RAAS. Interestingly, there are two additional pathways for RAAS activation. One is the physical compression of the kidney by adipose tissue in the visceral, retroperitoneal, and renal sinus sites. The other is the activation of the sympathetic nervous system (SNS) stimulated by high levels of BMI, especially an increase in renal sympathetic nerve activity (RSNA). Increased RSNA levels stimulate renin secretion and renal sodium reabsorption, promoting the development and maintenance of hypertension. Finally, there is increasing evidence that gut microbiota dysbiosis is associated with maintaining obesity with a high BMI. The close relationship between gut microbiota dysbiosis and lipid disorders and inflammatory responses is an important cause of hypertension. The gut microbiota can directly regulate the expression of fasting-induced adipocytokine (FIAL) genes in intestinal epithelial cells or reduce the production of FIAL by reducing the phosphorylation of adenosine monophosphate (AMP)-activated protein kinase. These changes lead to decreased hepatic fatty acid and adipose tissue oxidation and increased fatty acid intake, thereby contributing to the development of obesity and lipid metabolism disorders. Short-chain fatty acids (SCFAs) are the main metabolites of the intestinal flora. When the concentration of SCFAs is significantly increased (>0.9 mmol/L), this can antagonise orphan G-protein-coupled receptor (Gpr41) to inhibit vasodilation. This increases blood pressure. In addition, changes in the intestinal microbiota can also induce a systemic chronic inflammatory response, with a decrease in vasodilatory factors and an increase in vasoconstrictor factors. These changes increase systemic vascular resistance, which in turn leads to increased blood pressure.

The “Fluctuated (sharp-increase)” trajectory is characterised by a sharp increase in BMI over time from a normal baseline. Fan et al. (16) demonstrated that a series of pathophysiological and metabolic changes may occur during a rapid increase in BMI. These include aortic root enlargement, early vascular aging, and early endothelial dysfunction (41). These changes may have long-term and cumulative effects, such as the accumulation of visceral fat (42), inflammatory responses, activation of the SNS, and RAAS (43–46), which jointly lead to hypertension. Specifically, due to the rapid accumulation of visceral fat, on the one hand, adipose tissue rapidly produces a large amount of angiotensinogen, which activates RAAS. On the other hand, adipocytes recruit macrophages, induce M1 macrophage polarisation, and release many inflammatory cytokines that mediate IR. Both events can lead to a rapid increase in blood pressure.

The cause of the association between the “Fluctuated (elevated-decrease)” and hypertension may be related to the increase in sympathetic tone caused by elevated BMI, which may have a cumulative effect. Interestingly, we observed that the risk of hypertension was significantly reduced in children when their BMI dropped to a Stable normal, while adults remained at a higher risk of hypertension. A possible explanation is that the increase in sympathetic tone in children is mild and can be reversed after removing the risk factors. However, the cumulative damage caused by the increased sympathetic tone in adults was more severe. Therefore, the reduction in BMI did not significantly lower the sympathetic tone. In addition, arterial stiffness increases with age and peripheral vascular response to sympathetic tone decreases. Thus, the “Fluctuated (elevated-decrease)” trajectory in adults increases their risk of hypertension.

### 4.1. Strengths and limitations

The main strength of our study is that traditional meta-analysis and Bayesian network meta-analysis were used simultaneously. This provided more consequential information. Furthermore, this is the first systematic review and network meta-analysis to compare the prevalence of hypertension risk across different BMI trajectories. The approach reveals new insights that may influence hypertension management. We investigated the causes of heterogeneity across studies for each trajectory type through subgroup analyses. Many included studies have focused on selecting comparable “Stable normal” BMI trajectories as negative controls, providing reliable RR estimates.

However, this study had several limitations. First, in the traditional meta-analysis, heterogeneity was pronounced in the “Stable high” and “Fluctuated (sharp-increase)” trajectories. Second, the included studies for “Stable high” trajectories were limited and, thus, we could not determine the source of heterogeneity more accurately by means of meta-regression and subgroup analysis. Third, the included population was mostly Asian, with fewer participants from other regions. Further studies of hypertension risk factors in a more systematic manner will allow for a more detailed analysis.

## 5. Conclusion

“Stable high,” “Fluctuated (sharp increase),” and “Fluctuated (elevated-decrease)” trajectories were associated with increased hypertension risk. The “Stable low” trajectory was associated with a reduced risk of hypertension. The “Stable high” trajectory has the highest risk of hypertension. The findings highlight the necessity for different BMI control measures for hypertension prevention in children and adults and the importance of maintaining a stable normal BMI.

## Data availability statement

The original contributions presented in this study are included in the article/Supplementary material, further inquiries can be directed to the corresponding authors.

## Author contributions

LT and L-ZL conceived the study and analysed the data and wrote the draft of the manuscript. W-WY and F-FL searched the databases and checked them according to the eligibility criteria and exclusion criteria. X-CM and HQ helped develop search strategies. J-ML and F-FL contributed to reviewing or revising the manuscript. LT and C-GF were the guarantors of this study. All authors read and approved the final manuscript.
