# Supplementary material for: Association of body mass index trajectory and hypertension risk: A systematic review of cohort studies and network meta-analysis of 89,094 participants
Source: Front Cardiovasc Med. 2023 Jan 4;9:941341. doi: 10.3389/fcvm.2022.941341 (PMC9846820; doi:10.3389/fcvm.2022.941341)
Supplement: Supplementary file 1 [file Data_Sheet_1.docx]

**Association of body mass index trajectory and hypertension risk: A systematic review of cohort studies and network meta-analysis of 89,094 participants**

**Ling Tan^1^****^†^, Lin-zi Long^1†^, Xiao-chang Ma^1,2,^ Wen-wen Yang^1^, Fei-fei Liao^1,3^, Yu-xuan Peng^1,3^, Jie-ming Lu^1,3^, A-ling Shen^4^, Dong-qing An^5^, Hua Qu^1,2^, Chang-geng Fu^1,2^**

^1^Xiyuan Hospital, China Academy of Chinese Medical Sciences

^2^National Cardiovascular Clinical Medical Research Center of TCM

^3^Graduate School of Beijing University of Chinese Medicine

^4^Academy of Integrative Medicine, Fujian University of Traditional Chinese Medicine

^5^Affiliated Hospital of Traditional Chinese Medicine, Xinjiang Medical University

*** Correspondence:**

*E-mail addresses*: [hua_qu@yeah.net (Hua](mailto:hua_qu@yeah.net(Hua) Qu); [fucgbs@163.com](mailto:fucgbs@163.com) (Chang-geng Fu)

^†^These authors contributed equally to this work and share first authorship.

| **Section and**  **Supplementary Table 1. PRISMA 2020 checklist**  **Topic** | **Item**  **#** | **Checklist item** | **Location where item is reported** |
| --- | --- | --- | --- |
| **TITLE** | | |  |
| Title | 1 | Identify the report as a systematic review. | 1 |
| **ABSTRACT** | | |  |
| Abstract | 2 | See the PRISMA 2020 for Abstracts checklist. | 1-2 |
| **INTRODUCTION** | | |  |
| Rationale | 3 | Describe the rationale for the review in the context of existing knowledge. | 2 |
| Objectives | 4 | Provide an explicit statement of the objective(s) or question(s) the review addresses. | 2 |
| **METHODS** | | |  |
| Eligibility criteria | 5 | Specify the inclusion and exclusion criteria for the review and how studies were grouped for the syntheses. | 2 |
| Information sources | 6 | Specify all databases, registers, websites, organisations, reference lists and other sources searched or consulted to identify studies. Specify the date when each source was last searched or consulted. | 2 |
| Search strategy | 7 | Present the full search strategies for all databases, registers and websites, including any filters and limits used. | 2, Supplemental Table 2 |
| Selection process | 8 | Specify the methods used to decide whether a study met the inclusion criteria of the review, including how many reviewers screened each record and each report retrieved, whether they worked independently, and if applicable, details of automation tools used in the process. | 3 |
| Data collection process | 9 | Specify the methods used to collect data from reports, including how many reviewers collected data from each report, whether they worked independently, any processes for obtaining or confirming data from study investigators, and if applicable, details of automation tools used in the process. | 3 |
| Data items | 10a | List and define all outcomes for which data were sought. Specify whether all results that were compatible with each outcome domain in each study were sought (e.g. for all measures, time points, analyses), and if not, the methods used to decide which results to collect. | 3 |
|  | 10b | List and define all other variables for which data were sought (e.g. participant and intervention characteristics, funding sources). Describe any assumptions made about any missing or unclear information. | 3 |
| Study risk of bias assessment | 11 | Specify the methods used to assess risk of bias in the included studies, including details of the tool(s) used, how many reviewers assessed each study and whether they worked independently, and if applicable, details of automation tools used in the process4. | 3 |
| **Section and**  **Topic** | **Item**  **#** | **Checklist item** | **Location where item is reported** |
| Effect measures | 12 | Specify for each outcome the effect measure(s) (e.g. risk ratio, mean difference) used in the synthesis or presentation of results. | 3 |
| Synthesis methods | 13a | Describe the processes used to decide which studies were eligible for each synthesis (e.g. tabulating the study intervention characteristics and comparing against the planned groups for each synthesis (item #5)). | 3 |
|  | 13b | Describe any methods required to prepare the data for presentation or synthesis, such as handling of missing summary statistics, or data conversions. | 3 |
|  | 13c | Describe any methods used to tabulate or visually display results of individual studies and syntheses. | 3 |
|  | 13d | Describe any methods used to synthesize results and provide a rationale for the choice(s). If meta-analysis was performed, describe the model(s), method(s) to identify the presence and extent of statistical heterogeneity, and software package(s) used. | 3 |
|  | 13e | Describe any methods used to explore possible causes of heterogeneity among study results (e.g. subgroup analysis, meta-regression). | 3 |
|  | 13f | Describe any sensitivity analyses conducted to assess robustness of the synthesized results. | 4 |
| Reporting bias assessment | 14 | Describe any methods used to assess risk of bias due to missing results in a synthesis (arising from reporting biases). | 4 |
| Certainty assessment | 15 | Describe any methods used to assess certainty (or confidence) in the body of evidence for an outcome. | 4 |
| **RESULTS** | | |  |
| Study selection | 16a | Describe the results of the search and selection process, from the number of records identified in the search to the number of studies included in the review, ideally using a flow diagram. | 4 |
|  | 16b | Cite studies that might appear to meet the inclusion criteria, but which were excluded, and explain why they were excluded. | 4 |
| Study characterics | 17 | Cite each included study and present its characteristics. | 4-5 |
| Risk of bias in studies | 18 | Present assessments of risk of bias for each included study. | 10 |
| **Section and**  **Topic** | **Item**  **#** | **Checklist item** | **Location where item is reported** |
| Results of individual studies | 19 | For all outcomes, present, for each study: (a) summary statistics for each group (where appropriate) and (b) an effect estimate and its precision (e.g. confidence/credible interval), ideally using structured tables or plots. | 6-7 |
| Results of syntheses | 20a | For each synthesis, briefly summarise the characteristics and risk of bias among contributing studies. | 5 |
|  | 20b | Present results of all statistical syntheses conducted. If meta-analysis was done, present for each the summary estimate and its precision (e.g. confidence/credible interval) and measures of statistical heterogeneity. If comparing groups, describe the direction of the effect. | 8-9 |
|  | 20c | Present results of all investigations of possible causes of heterogeneity among study results. | 9,11-12 |
|  | 20d | Present results of all sensitivity analyses conducted to assess the robustness of the synthesized results. | 9 |
| Reporting biases | 21 | Present assessments of risk of bias due to missing results (arising from reporting biases) for each synthesis assessed. | 10 |
| Certainty of evidence | 22 | Present assessments of certainty (or confidence) in the body of evidence for each outcome assessed. | 10 |
| **DISCUSSION** | | |  |
| Discussion | 23a | Provide a general interpretation of the results in the context of other evidence. | 10, 14-15 |
|  | 23b | Discuss any limitations of the evidence included in the review. | 15 |
|  | 23c | Discuss any limitations of the review processes used. | 15 |
|  | 23d | Discuss implications of the results for practice, policy, and future research. | 15 |
| **OTHER INFORMATION** | | |  |
| Registration and protocal | 24a | Provide registration information for the review, including register name and registration number, or state that the review was not registered. | 2 |
|  | 24b | Indicate where the review protocol can be accessed, or state that a protocol was not prepared. | 2 |
|  | 24c | Describe and explain any amendments to information provided at registration or in the protocol. | 2 |
| Support | 25 | Describe sources of financial or non-financial support for the review, and the role of the funders or sponsors in the review. | 16 |
| Competing interests | 26 | Declare any competing interests of review authors. | 16 |
| **Section and**  **Topic** | **Item**  **#** | **Checklist item** | **Location where item is reported** |
| Availability of  data, code and  other materials | 27 | Report which of the following are publicly available and where they can be found: template data collection forms; data extracted from included studies; data used for all analyses; analytic code; any other materials used in the review. | 28 |

**Supplementary Table 2. Search strategy**

| **A: Search strategy in MEDLINE** | |
| --- | --- |
| **#** | Query |
| 1# | body mass index trajectory [Title/Abstract] OR body mass index change [Title/Abstract] OR quetelet index trajectory [Title/Abstract] OR quetelet index change [Title/Abstract] OR body size trajectory [Title/Abstract] OR body size change [Title/Abstract] OR body height trajectory [Title/Abstract] OR body height change [Title/Abstract] OR body weight trajectory [Title/Abstract] OR body weight change [Title/Abstract] OR body adiposity index trajectory [Title/Abstract] OR body adiposity index change [Title/Abstract] OR body shape index trajectory [Title/Abstract] OR body shape index change [Title/Abstract] OR body fat trajectory [Title/Abstract] OR body fat change [Title/Abstract] OR fat mass trajectory [Title/Abstract] OR fat mass change [Title/Abstract] OR waist circumference trajectory [Title/Abstract] OR waist circumference change [Title/Abstract] OR waist-height ratio trajectory [Title/Abstract] OR waist-height ratio change [Title/Abstract] OR waist-hip ratio trajectory [Title/Abstract] OR waist-hip ratio change [Title/Abstract] |
| 2# | blood pressure, high [Title/Abstract] OR blood pressures, high [Title/Abstract] OR high blood pressure [Title/Abstract] OR high blood pressures [Title/Abstract] OR hypertens*[Title/Abstract] OR idiopathic hypertension [Title/Abstract] OR essential hypertension [Title/Abstract] OR elevat* blood pressure [Title/Abstract] OR raised blood pressure [Title/Abstract] OR excessive blood pressure [Title/Abstract] OR elevat* diastolic blood pressure [Title/Abstract] OR elevat* systolic blood pressure [Title/Abstract] OR elevat* arterial blood pressure [Title/Abstract] OR high diastolic blood pressure [Title/Abstract] OR high systolic blood pressure [Title/Abstract] OR high arterial blood pressure [Title/Abstract] OR raised diastolic blood pressure [Title/Abstract] OR raised systolic blood pressure [Title/Abstract] OR raised arterial blood pressure [Title/Abstract] OR excessive diastolic blood pressure [Title/Abstract] OR excessive systolic blood pressure [Title/Abstract] OR excessive arterial blood pressure [Title/Abstract] |
| 3# | Observational stud*[Title/Abstract] OR cohort*[Title/Abstract] OR cohort stud*[Title/Abstract] OR longitudinal[Title/Abstract] OR longitudinal stud*[Title/Abstract] OR prospective[Title/Abstract] OR prospective stud*[Title/Abstract] OR follow-up stud* |
| 4# | 1# and 2# and 3# |
| **B: Search strategy in Embase** | |
| **#** | Query |
| 1# | ‘body mass index trajectory’:ab,ti OR ‘body mass index change’: ab,ti OR ‘quetelet index trajectory’:ab,ti OR ‘quetelet index change’: ab,ti OR ‘body size trajectory’: ab,ti OR ‘body size change’: ab,ti OR ‘body height trajectory’: ab,ti OR ‘body height change’ :ab,ti OR ‘body weight trajectory’: ab,ti OR ‘body weight change’:ab,ti OR ‘body adiposity index trajectory’: ab,ti OR ‘body adiposity index change’: ab,ti OR ‘body shape index trajectory’:ab,ti OR ‘body shape index change’:ab,ti OR ‘body fat trajectory’: ab,ti OR ‘body fat change’:ab,ti OR ‘fat mass trajectory’: ab,ti OR ‘fat mass change’:ab,ti OR ‘waist circumference trajectory’:ab,ti OR ‘waist circumference change’:ab,ti OR ‘waist-height ratio trajectory’:ab,ti OR ‘waist-height ratio change’:ab,ti OR ‘waist-hip ratio trajectory’:ab,ti OR ‘waist-hip ratio change’:ab,ti |
| 2# | ‘blood pressure, high’:ab,ti OR ‘blood pressures, high’:ab,ti OR ‘high blood pressure’:ab,ti OR ‘high blood pressures’:ab,ti OR ‘hypertens*’:ab,ti OR ‘idiopathic hypertension’: ab,ti OR ‘essential hypertension’:ab,ti OR ‘elevat* blood pressure’:ab,ti OR ‘raised blood pressure’: ab,ti OR ‘excessive blood pressure’:ab,ti OR ‘elevat* diastolic blood pressure’: ab,ti OR ‘elevat* systolic blood pressure’:ab,ti OR ‘elevat* arterial blood pressure’: ab,ti OR ‘high diastolic blood pressure’:ab,ti OR ‘high systolic blood pressure’: ab,ti OR ‘high arterial blood pressure’:ab,ti OR ‘raised diastolic blood pressure’:ab,ti OR ‘raised systolic blood pressure’:ab,ti OR ‘raised arterial blood pressure’:ab,ti OR ‘excessive diastolic blood pressure’:ab,ti OR ‘excessive systolic blood pressure’:ab,ti OR ‘excessive arterial blood pressure’:ab,ti |
| 3# | ‘Observational stud*’:ab,ti OR ‘cohort*’:ab,ti OR ‘cohort stud*’:ab,ti OR ‘longitudinal’:ab,ti OR ‘longitudinal stud*’:ab,ti OR ‘prospective’:ab,ti OR ‘prospective stud*’:ab,ti OR ‘follow-up stud*’:ab,ti |
| 4# | 1# and 2# and 3# |
| **C: Search strategy in Cochrane Library** | |
| **#** | Query |
| 1# | (body mass index trajectory): ti,ab,kw OR (body mass index change): ti,ab,kw OR (quetelet index trajectory): ti,ab,kw OR (quetelet index change): ti,ab,kw OR (body size trajectory) : ti,ab,kw OR (body size change): ti,ab,kw OR (body height trajectory): ti,ab,kw OR (body height change): ti,ab,kw OR (body weight trajectory): ti,ab,kw OR (body weight change) : ti,ab,kw OR (body adiposity index trajectory): ti,ab,kw OR (body adiposity index change) : ti,ab,kw OR (body shape index trajectory): ti,ab,kw OR (body shape index change) : ti,ab,kw OR (body fat trajectory): ti,ab,kw OR (body fat change): ti,ab,kw OR (fat mass trajectory) : ti,ab,kw OR (fat mass change): ti,ab,kw OR (waist circumference trajectory): ti,ab,kw OR (waist circumference change): ti,ab,kw OR (waist-height ratio trajectory): ti,ab,kw OR (waist-height ratio change): ti,ab,kw OR (waist-hip ratio trajectory): ti,ab,kw OR (waist-hip ratio change): ti,ab,kw |
| 2# | (blood pressure, high): ti,ab,kw OR (blood pressures, high): ti,ab,kw OR (high blood pressure): ti,ab,kw OR (high blood pressures): ti,ab,kw OR (hypertens*): ti,ab,kw OR (idiopathic hypertension): ti,ab,kw OR (essential hypertension): ti,ab,kw OR (elevat* blood pressure): ti,ab,kw OR (raised blood pressure): ti,ab,kw OR (excessive blood pressure): ti,ab,kw OR (elevat* diastolic blood pressure): ti,ab,kw OR (elevat* systolic blood pressure) : ti,ab,kw OR (elevat* arterial blood pressure): ti,ab,kw OR (high diastolic blood pressure): ti,ab,kw OR (high systolic blood pressure): ti,ab,kw OR (high arterial blood pressure): ti,ab,kw OR (raised diastolic blood pressure): ti,ab,kw OR (raised systolic blood pressure): ti,ab,kw OR (raised arterial blood pressure): ti,ab,kw OR (excessive diastolic blood pressure): ti,ab,kw OR (excessive systolic blood pressure): ti,ab,kw OR (excessive arterial blood pressure): ti,ab,kw |
| 3# | (Observational stud*): ti,ab,kw OR (cohort*): ti,ab,kw OR (cohort stud*): ti,ab,kw OR (longitudinal): ti,ab,kw OR (longitudinal stud*): ti,ab,kw OR (prospective): ti,ab,kw OR (prospective stud*): ti,ab,kw OR (follow-up stud*): ti,ab,kw |
| 4# | 1# and 2# and 3# |
| **D: Search strategy in Scopus** | |
| **#** | Query |
| 1# | TITLE-ABS-KEY ( “body mass index trajectory”) OR TITLE-ABS-KEY (“body mass index change”) OR TITLE-ABS-KEY (“quetelet index trajectory”) OR TITLE-ABS-KEY (“quetelet index change”) OR TITLE-ABS-KEY (“body size trajectory”) OR TITLE-ABS-KEY (“body size change”) OR TITLE-ABS-KEY (“body height trajectory”) OR TITLE-ABS-KEY (“body height change”) OR TITLE-ABS-KEY (“body weight trajectory”) OR TITLE-ABS-KEY (“body weight change”) OR TITLE-ABS-KEY(“body adiposity index trajectory”) OR TITLE-ABS-KEY(“body adiposity index change”) OR TITLE-ABS-KEY (“body shape index trajectory”) OR TITLE-ABS-KEY (“body shape index change”) OR TITLE-ABS-KEY (“body fat trajectory”) OR TITLE-ABS-KEY (“body fat change”) OR TITLE-ABS-KEY (“fat mass trajectory”) OR TITLE-ABS-KEY (“fat mass change”) OR TITLE-ABS-KEY (“waist circumference trajectory”) OR TITLE-ABS-KEY (“waist circumference change”) OR TITLE-ABS-KEY(“waist-height ratio trajectory”) OR TITLE-ABS-KEY (“waist-height ratio change”) OR TITLE-ABS-KEY(“waist-hip ratio trajectory”) OR TITLE-ABS-KEY ( “waist-hip ratio change”) |
| 2# | TITLE-ABS-KEY (“hypertension”) OR TITLE-ABS-KEY (“blood pressure, high”) OR TITLE-ABS-KEY (“blood pressures, high”) OR TITLE-ABS-KEY(“high blood pressure”) OR TITLE-ABS-KEY(“high blood pressures”) OR TITLE-ABS-KEY(“hypertens*”) OR TITLE-ABS-KEY(“idiopathic hypertension”) OR TITLE-ABS-KEY(“essential hypertension”) OR TITLE-ABS-KEY(“elevatory blood pressure”) OR TITLE-ABS-KEY (“raised blood pressure”) OR TITLE-ABS-KEY (“excessive blood pressure”) OR TITLE-ABS-KEY(“elevatory diastolic blood pressure”) OR TITLE-ABS-KEY (“elevatory systolic blood pressure”) OR TITLE-ABS-KEY (“elevatory arterial blood pressure”) OR TITLE-ABS-KEY (“high diastolic blood pressure”) OR TITLE-ABS-KEY (“high systolic blood pressure”) OR TITLE-ABS-KEY(“high arterial blood pressure”) OR TITLE-ABS-KEY (“raised diastolic blood pressure”) OR TITLE-ABS-KEY (“raised systolic blood pressure”) OR TITLE-ABS-KEY (“raised arterial blood pressure”) OR TITLE-ABS-KEY(“excessive diastolic blood pressure”) OR TITLE-ABS-KEY (“excessive systolic blood pressure”) OR TITLE-ABS-KEY (“excessive arterial blood pressure”) |
| 3# | TITLE-ABS-KEY(“Observational stud*”) OR TITLE-ABS-KEY (“cohort*”) OR TITLE-ABS-KEY(“cohort stud*”) OR TITLE-ABS-KEY (“longitudinal”) OR TITLE-ABS-KEY (“longitudinal stud*”) OR TITLE-ABS-KEY (“prospective”) OR TITLE-ABS-KEY (“prospective stud*”) OR TITLE-ABS-KEY ( “follow-up stud*”) |
| 4# | 1# and 2# and 3# |
| **E:** **Search strategy in Web of Science** | |
| 1# | TS=(body mass index trajectory OR body mass index change OR quetelet index trajectory OR quetelet index change OR body size trajectory OR body size change OR body height trajectory OR body height change OR body weight trajectory OR body weight change OR body adiposity index trajectory OR body adiposity index change OR body shape index trajectory OR body shape index change OR body fat trajectory OR body fat change OR fat mass trajectory OR fat mass change OR waist circumference trajectory OR waist circumference change OR waist-height ratio trajectory OR waist-height ratio change OR waist-hip ratio trajectory OR waist-hip ratio change) |
| 2# | TS=(hypertension OR blood pressure, high OR high blood pressure OR blood pressures, high OR high blood pressures OR hypertens* OR idiopathic hypertension OR essential hypertension OR elevat* blood pressure OR elevat* systolic blood pressure OR raised blood pressure OR excessive blood pressure OR excessive blood pressure OR elevat* diastolic blood pressure OR high diastolic blood pressure OR elevat* arterial blood pressure OR high systolic blood pressure OR high arterial blood pressure OR raised diastolic blood pressure OR raised systolic blood pressure OR raised arterial blood pressure OR excessive diastolic blood pressure OR excessive systolic blood pressure OR excessive arterial blood pressure) |
| 3# | TS=(Observational stud* OR cohort* OR cohort stud* OR longitudinal OR longitudinal stud* OR prospective OR prospective stud* OR follow-up stud*) |
| 4# | 1# and 2# and 3# |

**Supplementary Table 3. Evaluation of local inconsistency for direct and indirect comparisons using node-split model.**

| **Name** | **Direct Effect** | | **Indirect Effect** | | **Overall** | | **P-Value** |
| --- | --- | --- | --- | --- | --- | --- | --- |
| **A,B** | 0.0284837 | 0.3373684 | -1.089848 | 0.6795004 | 1.118332 | 0.7480262 | 0.135 |
| **A,D** | 0 .4825778 | 0.820253 | 0.7333531 | 0.4466837 | -0.2507753 | 0.9393694 | 0.789 |
| **A,E** | 0.3707947 | 0.3717866 | 1.331222 | 0.6880083 | -0.9604271 | 0.8064972 | 0.234 |
| **B,C** | 0.2928055 | 0.2473767 | 0.0869463 | 0.5882546 | 0.2058592 | 0.6164788 | 0.738 |
| **B,D** | 0 .8249175 | 0.2782698 | 1.092155 | 0.6619053 | -0.2672375 | 0.7070956 | 0.705 |
| **B,E** | 0.7380913 | 0.1545764 | 2.576586 | 0.9649444 | -1.838495 | 0.9739088 | 0.059 |
| **C,D** | 0.074921 | 0.5618024 | 0.8344288 | 0.3822245 | -0.7595078 | 0.6794839 | 0.264 |
| **C,E** | 0 .613181 | 0.2640012 | 0.0382403 | 0.5907842 | 0.5749408 | 0.6375694 | 0.367 |
| **D,E** | 0.0158358 | 0.3953693 | -0.1608747 | 0.4165963 | 0.1767105 | 0.5835465 | 0.762 |

A: Stable low; B: Stable normal; C: Fluctuated(elevated-decrease); D: Stable high; E: Fluctuated(sharp-increase)

**Supplementary Table 4. GRADE assessment of the certainty of the evidence for direct comparisons of hypertension incidence between various BMI trajectories.**

| **Outcomes** | **Intervention and Comparison intervention** | **Illustrative comparative risks* (95% CI)** | | **Relative effect (95% CI)** | **No of Participants (studies)** | **Quality of the evidence (GRADE)** |
| --- | --- | --- | --- | --- | --- | --- |
|  |  | **Assumed risk** | **Corresponding risk** |  |  |  |
|  |  | **With comparator** | **With intervention** |  |  |  |
|  | "Stable low" BMI trajectory/"Stable normal" BMI trajectory | **Study population** | | **RR 0.83**  (0.45 to 1.53) | 15905 (3 studies) | ⊕⊝⊝⊝ **very low** |
|  |  | **307 per 1000** | **255 per 1000** (138 to 470) |  |  |  |
|  |  | **Moderate** | |  |  |  |
|  |  |  |  |  |  |  |
|  | "Stable low" BMI trajectory/"Stable high" BMI trajectory | **Study population** | | **RR 1.97**  (0.93 to 4.18) | 255 (1 study) | ⊕⊝⊝⊝ **very low** |
|  |  | **400 per 1000** | **788 per 1000** (372 to 1000) |  |  |  |
|  |  | **Moderate** | |  |  |  |
|  |  |  |  |  |  |  |
|  | "Fluctuated(sharp-increase)" BMI trajectory/"Stable low" BMI trajectory | **Study population** | | **RR 1.83**  (0.98 to 3.41) | 10521 (3 studies) | ⊕⊝⊝⊝ **very low** |
|  |  | **182 per 1000** | **333 per 1000** (178 to 620) |  |  |  |
|  |  | **Moderate** | |  |  |  |
|  |  |  |  |  |  |  |
|  | "Fluctuated(elevated-decrease)" BMI trajectory/"Stable normal" BMI trajectory | **Study population** | | **RR 1.31**  (0.83 to 2.05) | 24444 (8 studies) | ⊕⊝⊝⊝ **very low** |
|  |  | **346 per 1000** | **454 per 1000** (288 to 710) |  |  |  |
|  |  | **Moderate** | |  |  |  |
|  |  |  |  |  |  |  |
|  | "Stable high" BMI trajectory/"Stable normal" BMI trajectory | **Study population** | | **RR 2.37**  (1.44 to 3.89) | 23807 (5 studies) | ⊕⊝⊝⊝ **very low** |
|  |  | **458 per 1000** | **1000 per 1000** (659 to 1000) |  |  |  |
|  |  | **Moderate** | |  |  |  |
|  |  |  |  |  |  |  |
|  | "Fluctuated(sharp-increase)" BMI trajectory/"Stable normal" BMI trajectory | **Study population** | | **RR 2.20**  (1.60 to 3.03) | 58117 (16 studies) | ⊕⊝⊝⊝ **very low** |
|  |  | **320 per 1000** | **703 per 1000** (512 to 969) |  |  |  |
|  |  | **Moderate** | |  |  |  |
|  |  |  |  |  |  |  |
|  | "Stable high" BMI trajectory/"Fluctuated(elevated-decrease)" BMI trajectory | **Study population** | | **RR 1.81**  (0.97 to 3.39) | 11223 (1 study) | ⊕⊝⊝⊝ **very low** |
|  |  | **502 per 1000** | **908 per 1000** (487 to 1000) |  |  |  |
|  |  | **Moderate** | |  |  |  |
|  |  |  |  |  |  |  |
|  | "Fluctuated(sharp-increase)" BMI trajectory/"Fluctuated(elevated-decrease)" BMI trajectory | **Study population** | | Not estimable | 24969 (8 studies) | ⊕⊝⊝⊝ **very low** |
|  |  | **377 per 1000** | **0 per 1000** (0 to 0) |  |  |  |
|  |  | **Moderate** | |  |  |  |
|  | "Stable high" BMI trajectory/"Persistent increaing" BMI trajectory | **Study population** | | **RR 0.93**  (0.54 to 1.60) | 16890 (3 studies) | ⊕⊝⊝⊝ **very low** |
|  |  | **500 per 1000** | **465 per 1000** (270 to 799) |  |  |  |
|  |  | **Moderate** | |  |  |  |
|  |  |  |  |  |  |  |

**Supplementary Figure 1.**

**Meta-analysis results of the association between BMI trajectories and the risk of hypertension. (A) “Stable low” trajectory; (B) “Fluctuated(elevated-decrease)” trajectory; (C) “Fluctuated(sharp-increase)” trajectory; (D) “Stable high” trajectory.**

**
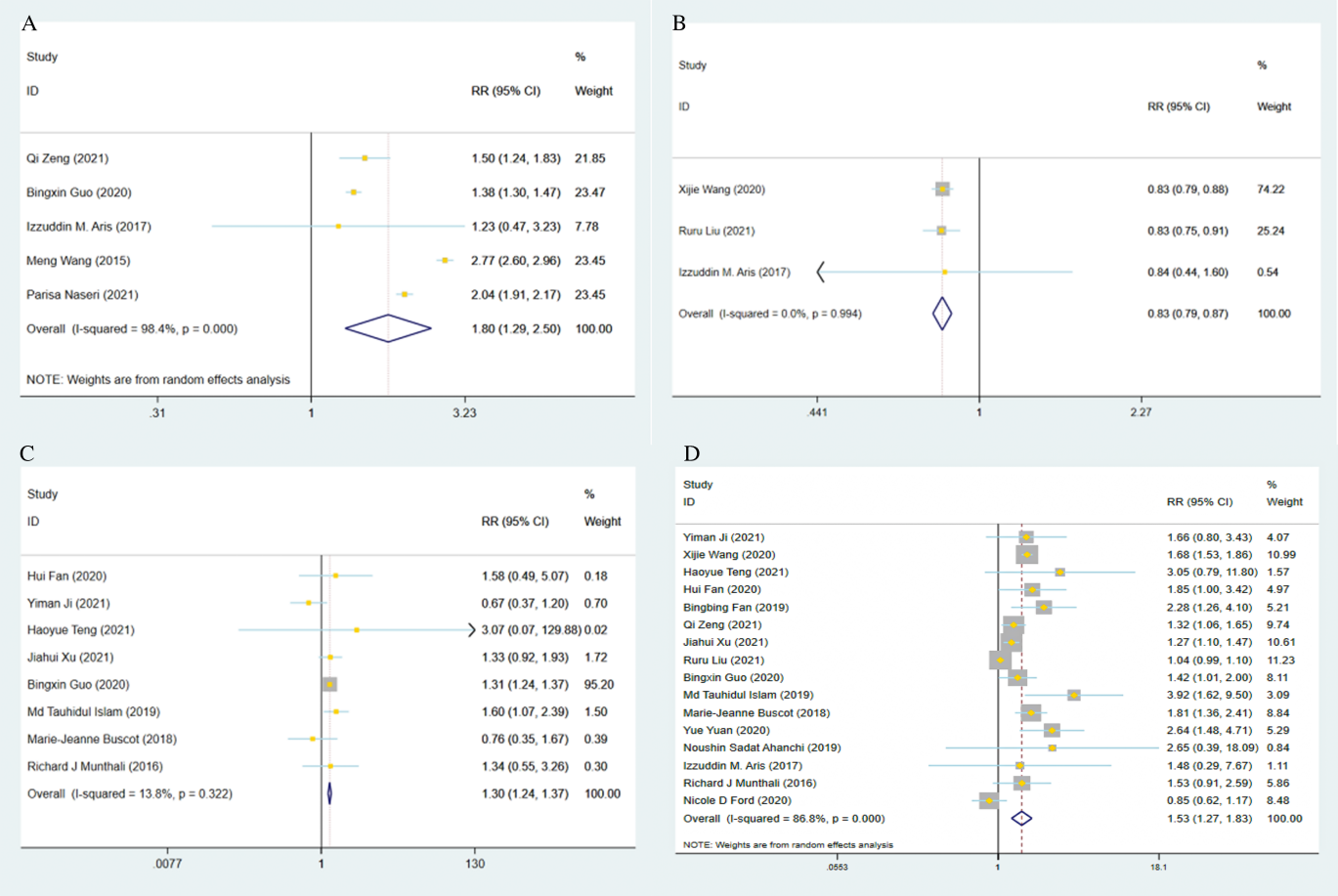
**

**Supplementary Figure 2.**

**Network evidence plot of included studies: the lines indicate direct comparisons between groups, and the size of the circled areas indicates the respective sample.**

**
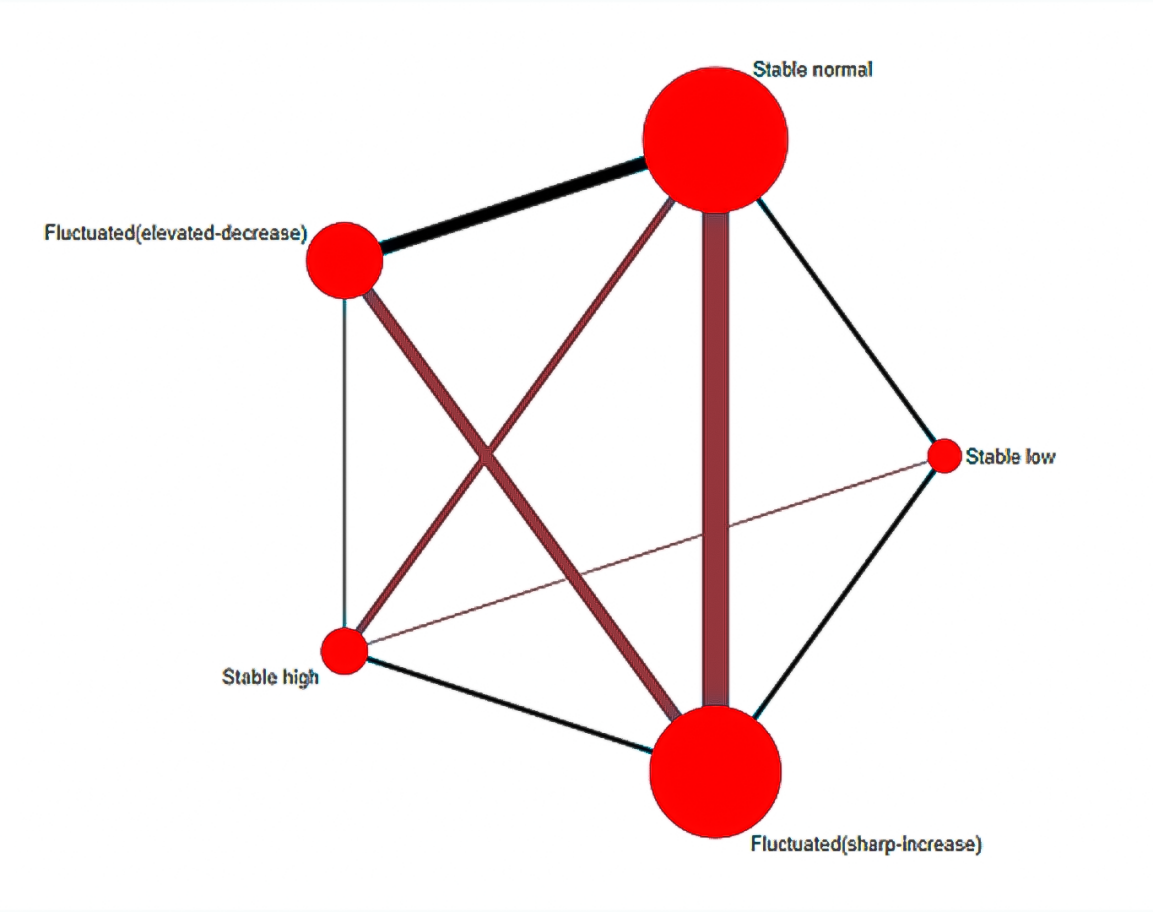
**

**Supplementary Figure 3.**

1. **Trajectory density graph of Markov chain on total effective rate.**
2. **B. Brooks-Gelman-Rubin diagnostic plot.**

**
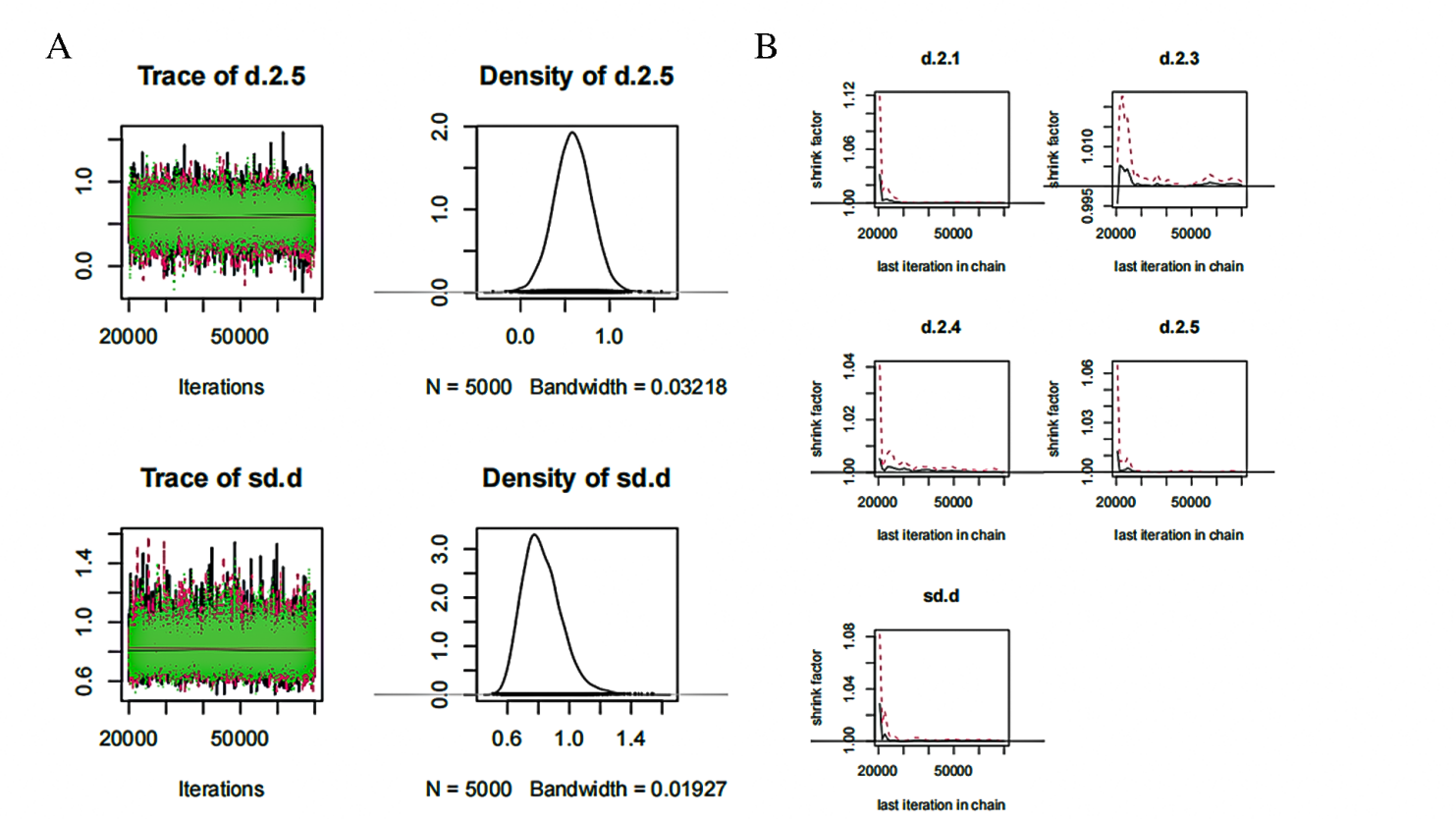
**

**Supplementary Figure 4.**

**SUCRA ranking plot: SURCA curve obtained by iterating 10,000 times.**

**
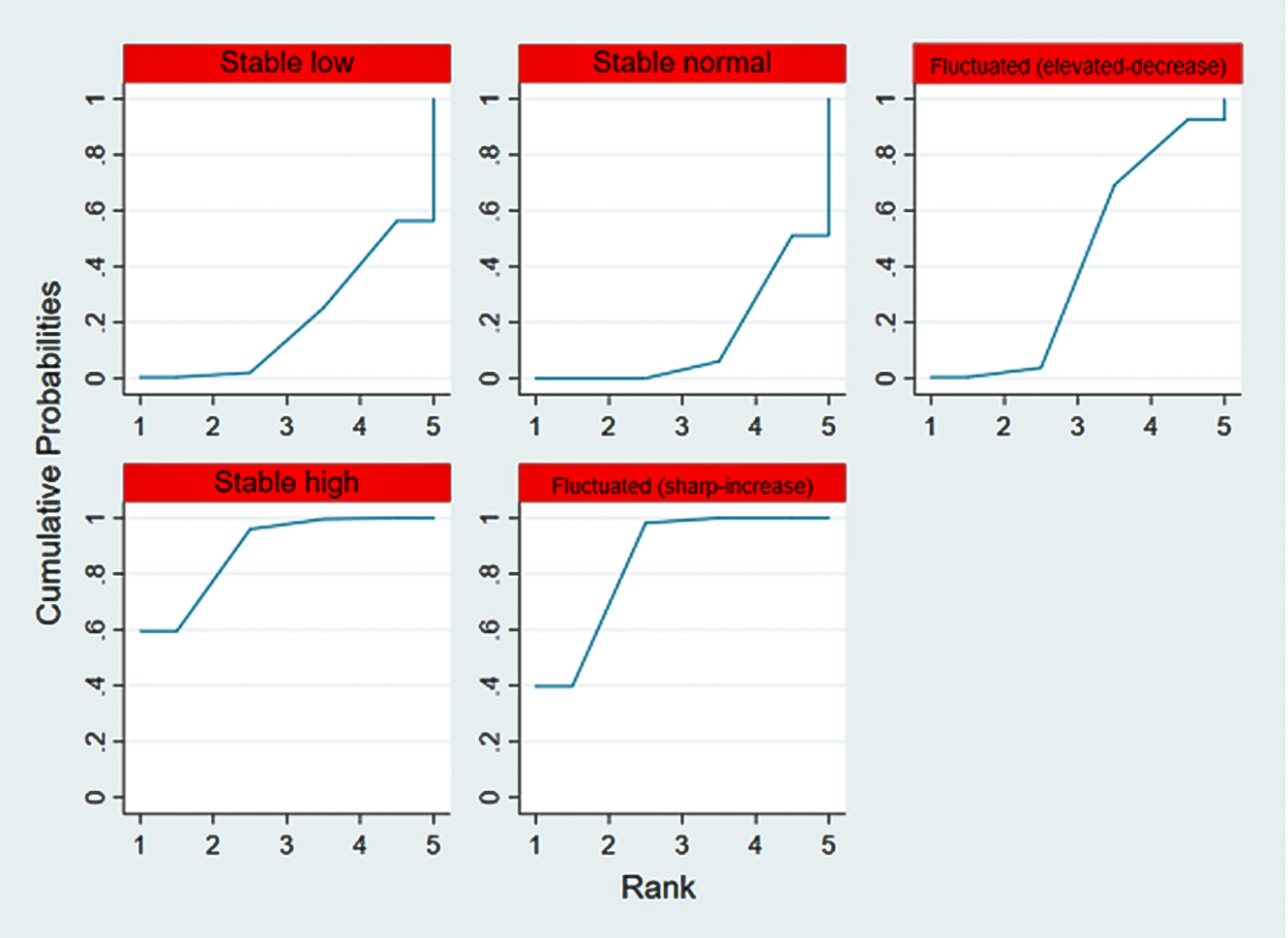
**

**Supplementary Figure 5.**

**Effect values for two-by-two comparisons between different BMI trajectories.**

| Fluctuated (sharp-increase) | 0.93 (0.54,1.60) | 1.69 (1.05,2.72) | 2.20 (1.60,3.03) | 1.83 (0.98,3.41) |
| --- | --- | --- | --- | --- |
| 1.08 (0.62,1.85) | Stable high | 1.81 (0.97,3.39) | 2.37 (1.44,3.89) | 1.97 (0.93,4.17) |
| 0.59 (0.37,0.96) | 0.55 (0.30,1.03) | Fluctuated (elevated-decrease) | 1.31 (0.83,2.05) | 1.09 (0.52,2.26) |
| 0.45 (0.33,0.63) | 0.42 (0.26,0.69) | 0.77 (0.49,1.20) | Stable normal | 0.83 (0.45,1.53) |
| 0.55 (0.29,1.02) | 0.51 (0.24,1.08) | 0.92 (0.44,1.92) | 1.20 (0.65,2.21) | Stable low |

**Supplementary Figure 6.**

**The results of meta-regression analysis of “Fluctuated(sharp-increase)” trajectory. (A) Follow-up time; (B) Sample size; (C) The number of BMI measurements; (D) Baseline systolic blood pressure; (E) Baseline diastolic blood pressure; (F) Baseline BMI values.**

**
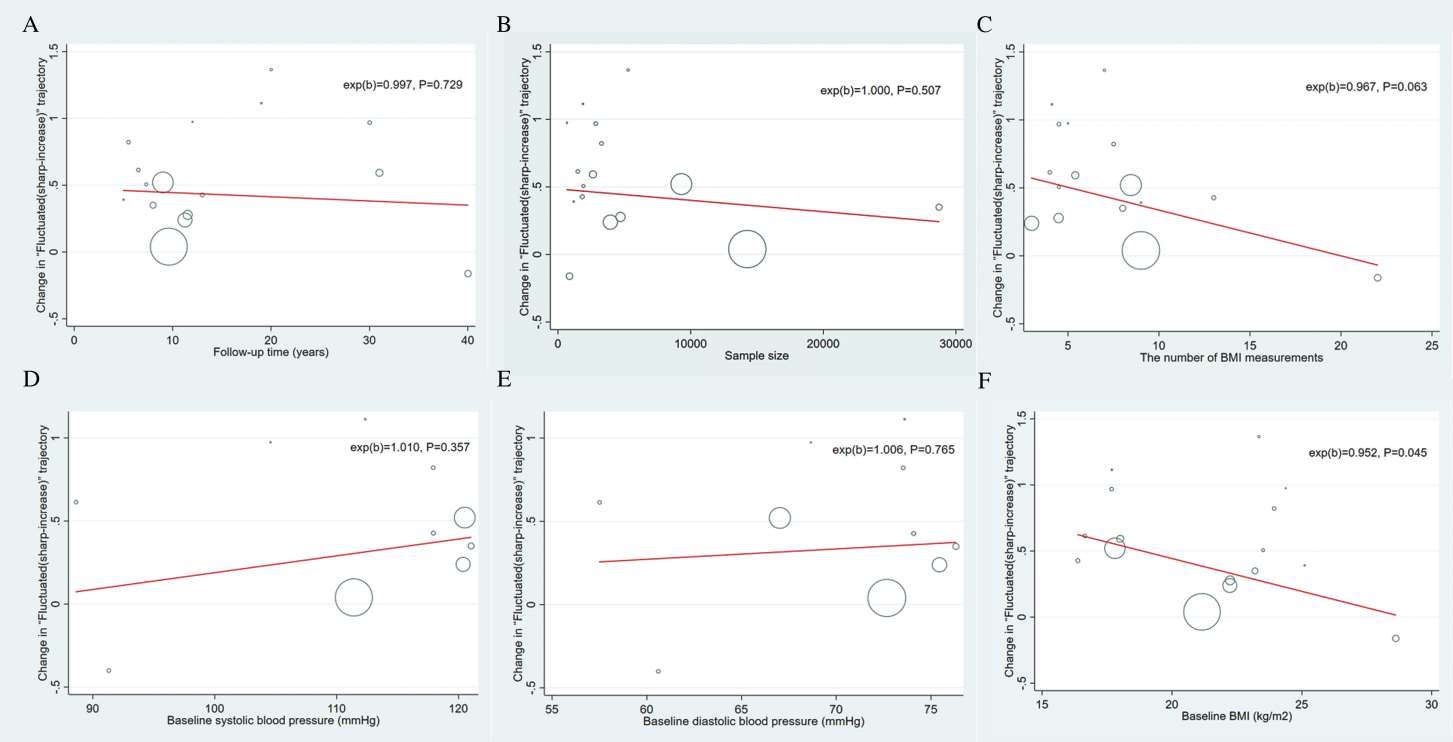
**

**Supplementary Figure 7.**

**The relationship between “Fluctuated(elevated-decrease)” BMI trajectories and the risk of hypertension based on subgroup analysis.**

**
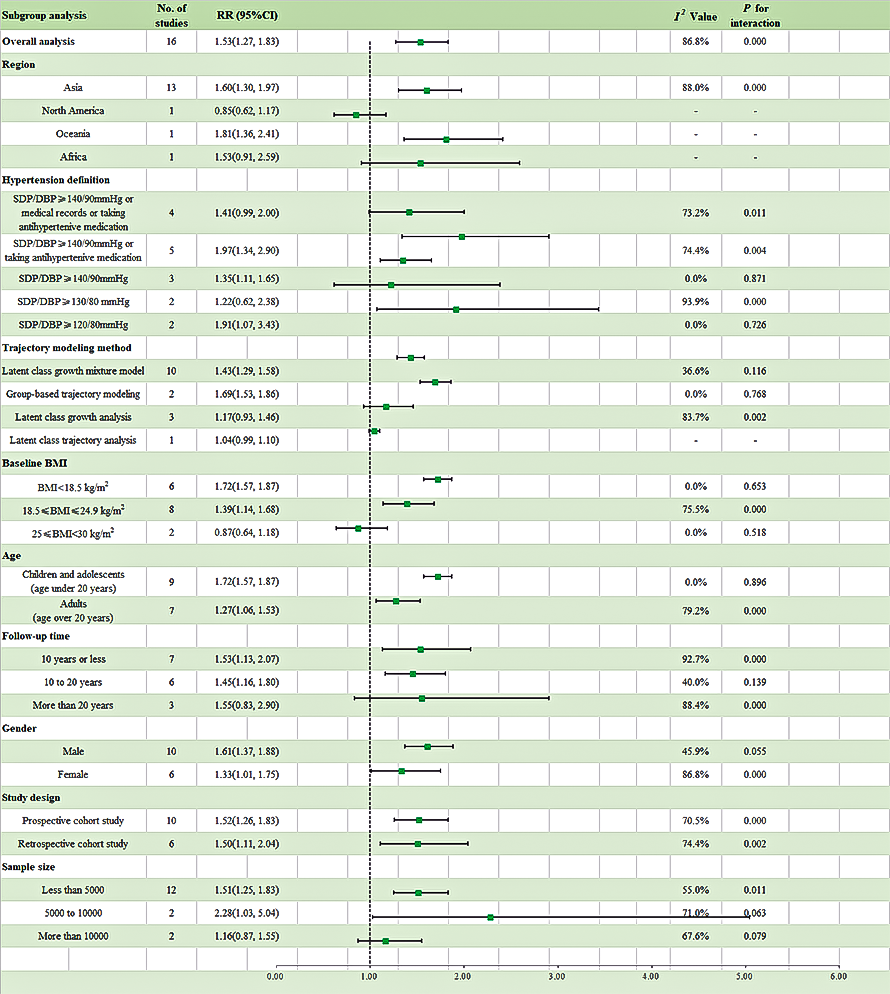
**

**Supplementary Figure 8.**

**The relationship between “Fluctuated(sharp-increase)” BMI trajectories and the risk of hypertension based on subgroup analysis.**

**
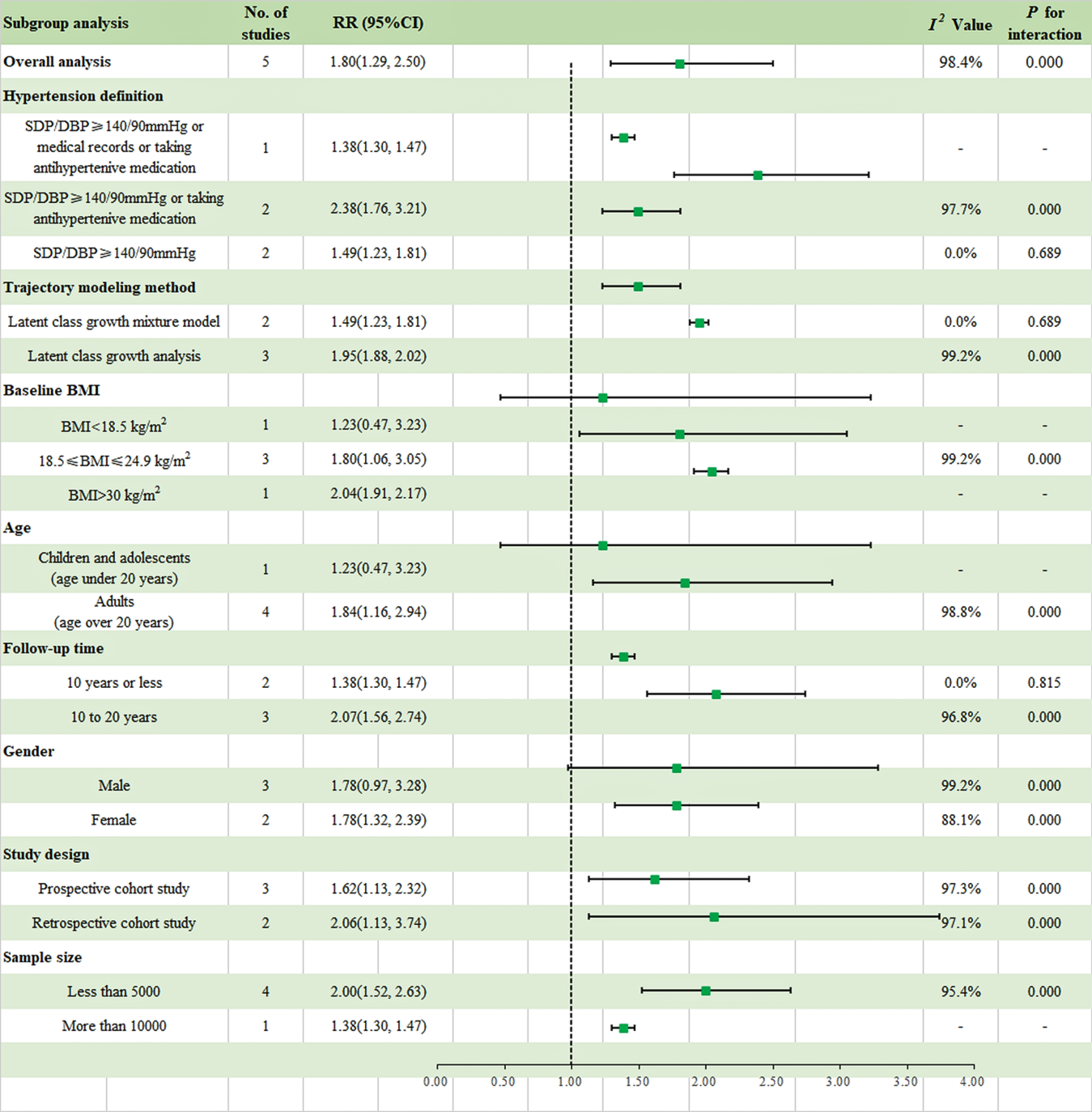
**

**Supplementary Figure 9.**

**The relationship between “Stable high” BMI trajectories and the risk of hypertension based on subgroup analysis.**

**
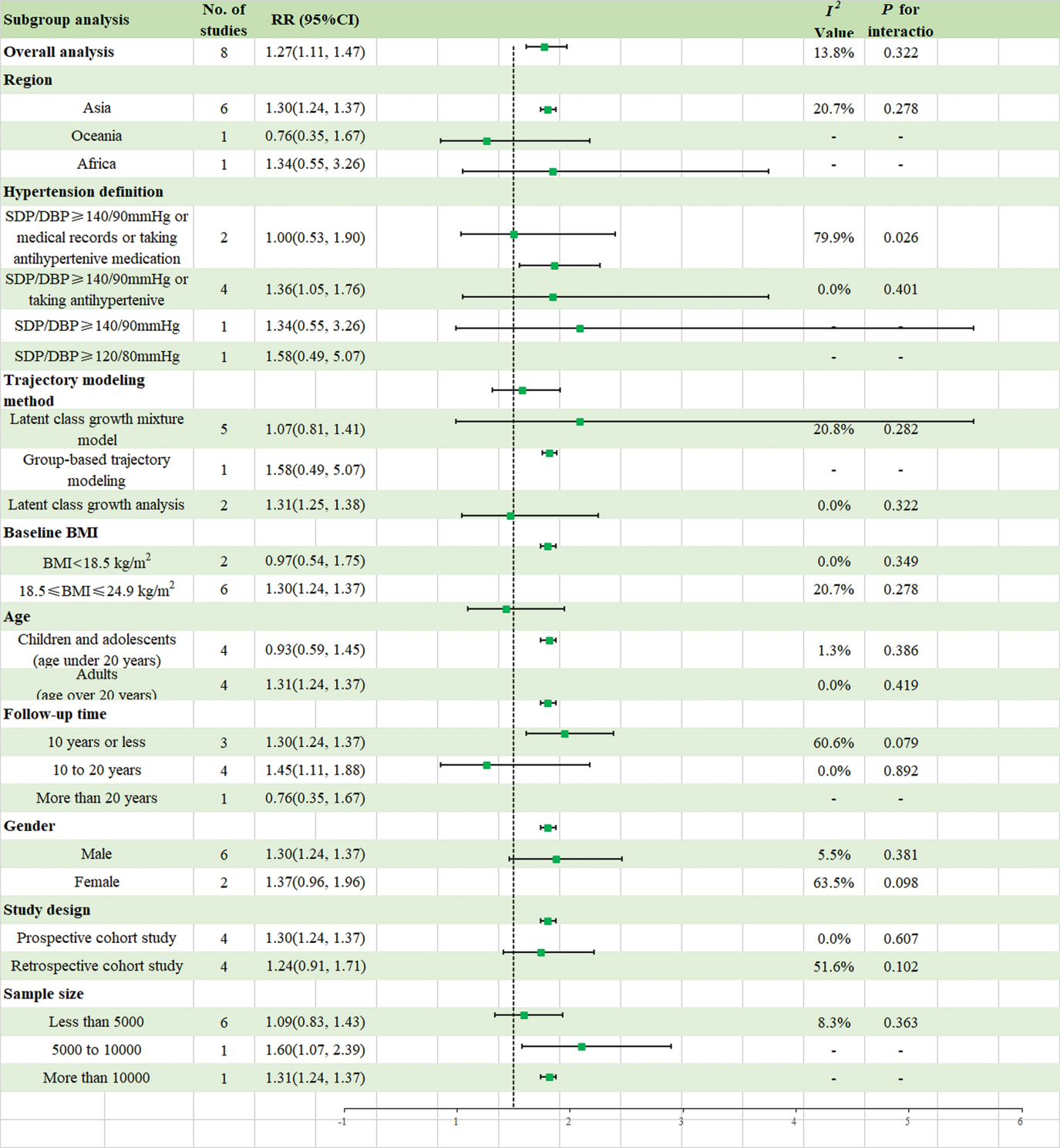
**

**Supplementary Figure 10.**

**Funnel plot of publication bias.**

**
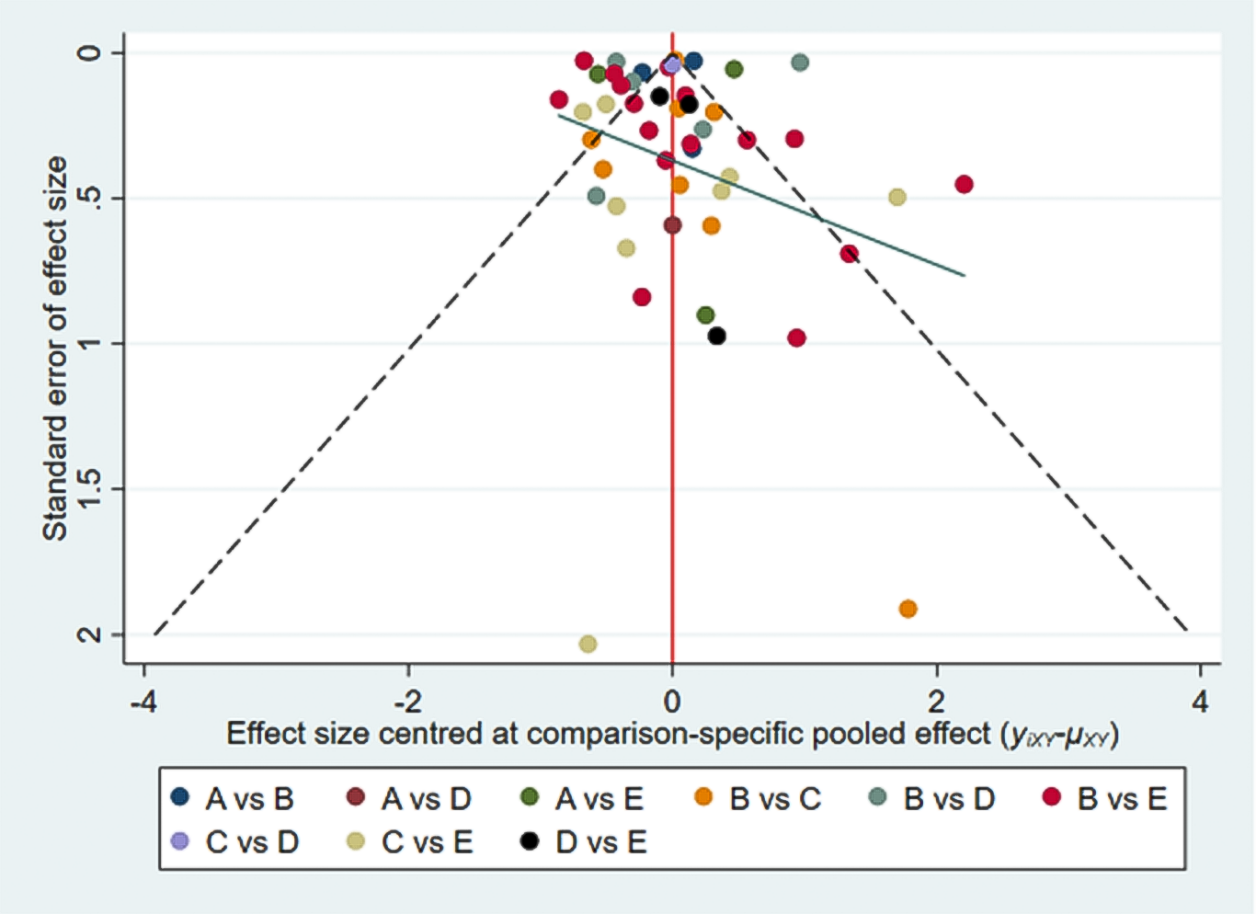
**

**Legend: B, stable normal; A, stable low; C, Fluctuated(elevated-decrease); D, stable high; E, Fluctuated(sharp-increase).**

**Supplementary Figure 11.**

**Prediction interval plot of the incidence of hypertension in each group.**

**
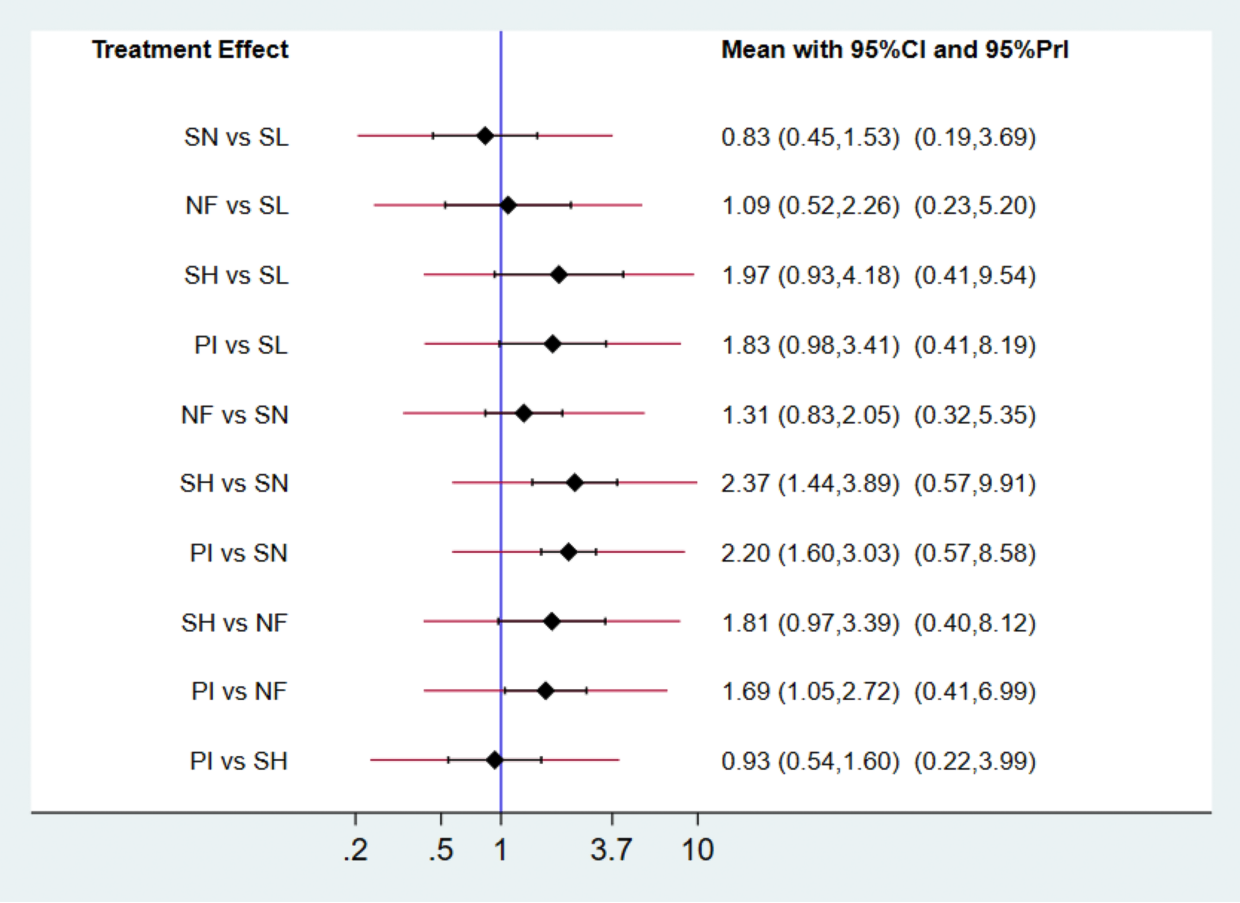
**

**Legend: SN, stable normal; SL, stable low; NF, Fluctuated(elevated-decrease); SH, stable high; PI, Fluctuated(sharp-increase).**
